# Body size and sequence of host colonisation predict the presence of acoustic signalling in beetles

**DOI:** 10.1038/s41598-024-66108-8

**Published:** 2024-07-05

**Authors:** Carol L. Bedoya, Eckehard G. Brockerhoff, Lawrence R. Kirkendall, Richard W. Hofstetter, Ximena J. Nelson

**Affiliations:** 1 School of Biological Sciences, University of Canterbury, Christchurch, Canterbury New Zealand; 2 Scion (New Zealand Forest Research Institute), Christchurch, Canterbury New Zealand; 3 Swiss Federal Research Institute WSL, Birmensdorf, Zurich, Switzerland; 4 Department of Biological Sciences, University of Bergen, Bergen, Vestland Norway; 5 School of Forestry, Northern Arizona University, Flagstaff, AZ USA; 6 Present Address: Atarau Sanctuary, Christchurch, Canterbury New Zealand

**Keywords:** Sexual selection, Entomology, Animal behaviour

## Abstract

Acoustic communication is widespread in beetles, is often sexually dimorphic, and plays a significant role in behaviours such as premating recognition, courtship, and copulation. However, the factors that determine the presence or absence of acoustic signalling in a given species remain unclear. We examined acoustic communication in bark beetles (Scolytinae) and pinhole borers (Platypodinae), which are two speciose groups with widespread sound production capabilities. We show that body size along with the sequence of host colonisation predict the presence of acoustic communication, and report, for the first time in the animal kingdom, a size limit—1.9 mm—below which acoustic signalling ceases to be present.

## Introduction

Constituting 41% of insects and 23% of all animals^1,2^, Coleoptera (beetles) is the most speciose animal order, yet beetle acoustic communication remains acutely understudied. Presence or absence of hearing organs, sound-producing organs, and acoustic signals remains uninvestigated for almost all beetle species^3–5^, obscuring the occurrence of widespread acoustic signalling patterns in the group. From the available information, it has been suggested that the varying roles of the sexes in host selection may determine the presence of acoustic communication^6,7^, although this hypothesis has never been tested on a large scale.

We used bark beetles (Scolytinae) and pinhole borers (Platypodinae), two related beetle subfamilies in the family Curculionidae that independently evolved acoustic communication^6,8^, to address two unresolved issues related to acoustic communication and sex-specific roles in beetles. Specifically, we considered (a) what factors determine the presence or absence of sound production in a species, and (b) which sex uses acoustic signalling. We report for the first time a critical body-size threshold for the loss of acoustic communication in an animal taxon. We also found that acoustic signalling is always present in the sex opposite to the one that first colonises the host.

With ca. 7500 described species between them^9^, bark beetles and pinhole borers, along with *Drosophila, Micronecta* water bugs, braconid wasps, whiteflies, and psyllids, are the smallest animals that use acoustic communication^6,10–14^. Bark beetles and pinhole borers breed in tunnels inside plant tissues (in most cases, in woody plants), where they use acoustic signals to communicate in different behavioural contexts, including species recognition, sexual selection, and distress^3,6,8^. The extreme morphological and behavioural similitudes between the two subfamilies^9^, including acoustic communication^6,8^, for which they arrived via different evolutionary paths^6,8,9^, might provide insights about the underlying mechanisms driving the evolution of acoustic communication in non-sister taxa.

## Results and discussion

We collected information related to the presence of acoustic communication, which sex stridulates, type of stridulatory organ, morphology, life history, behaviour, and phylogeny of bark beetles and pinhole borers, including details of 33% of the species which come from our own findings (Methods, Supplementary Table S1). Two clear patterns arise from our results: (i) there is a body size limit below which acoustic communication is not possible; and (ii) acoustic signalling is always present in the sex that does not colonise a host first (i.e., the second-arriving sex).

**Figure 1:**
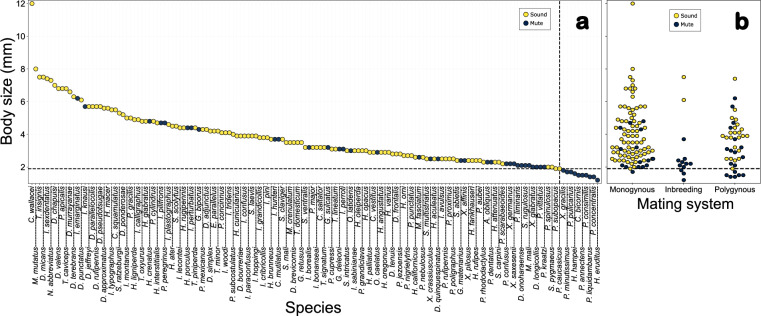
Body size and mating system of bark beetles and pinhole borers. (**A**) Body size (length) of the species where the presence or absence of acoustic signalling was reported. No species below 1.9 mm had sound production capabilities. (**B**) Body size vs mating system. All species that exclusively inbreed are mute. *Dendroctonus micans* and *D. punctatus*, the two outliers in the category 'Inbreeding', can both inbreed and outbreed.

**Figure 2:**
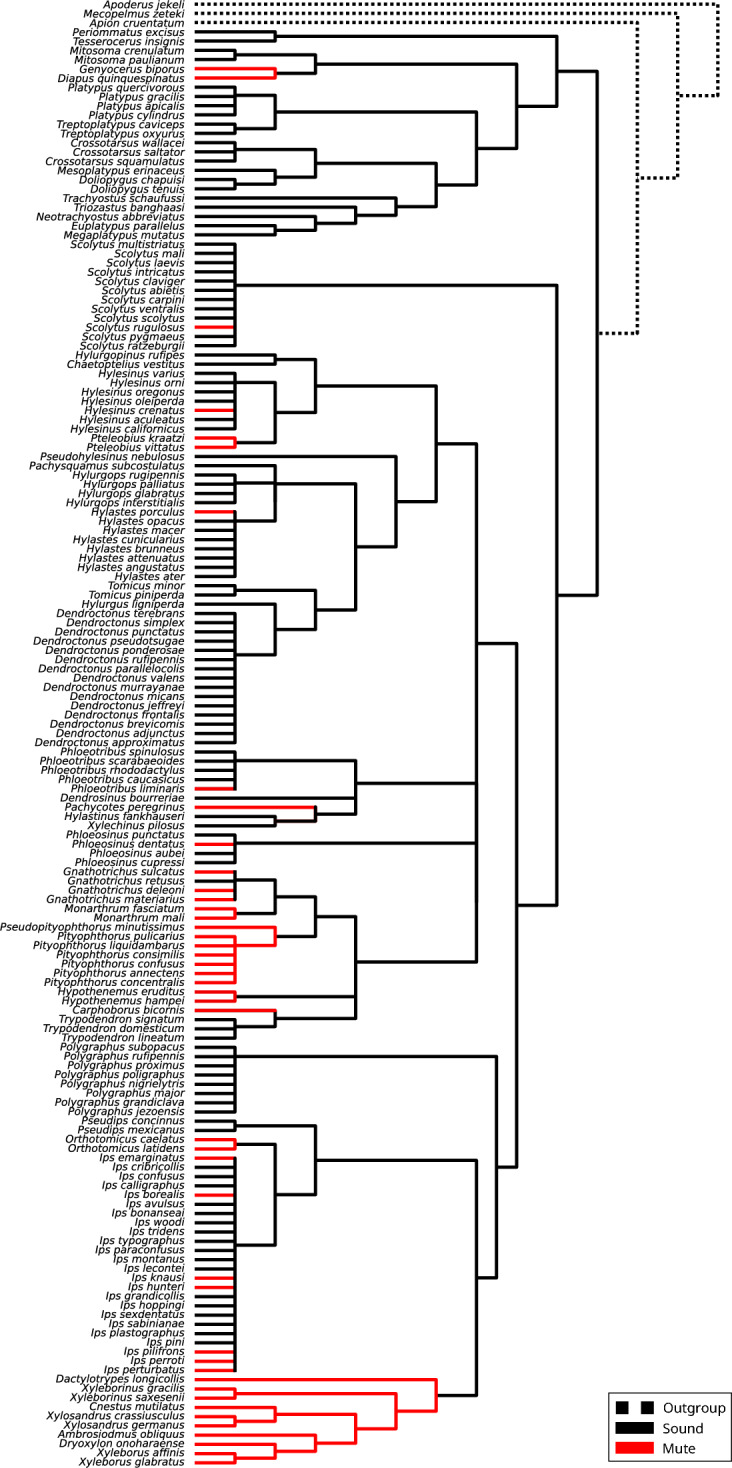
Cladogram of the studied species. Mute species are highlighted in red. Stridulating species are shown in black. Dotted lines indicate the outgroup.

**Figure 3:**
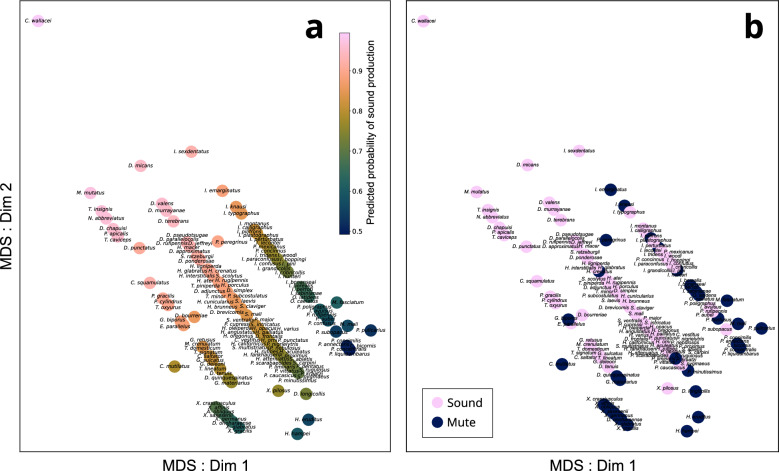
Logistic regression of the studied species. (**A**) Logistic regression of mating system and body size of Scolytinae and Platypodinae displayed on a multi-dimensional scaling (MDS) of life history variables (size, mating system, and feeding mode) and (**B**) ‘ground truth’ of the presence (sound) or absence (mute) of sound production. Mating system and body size predicted acoustic communication in 71% of the species.

In our review, no species below 1.9 mm in total length had sound production capabilities (pattern i) (Fig. 1A). A minimum body size limit for sound production is expected, as muscle power and the distance over which sounds can be perceived are proportional to the mass of the individual^12^. However, this is the first time a biological limit has been established. Our data also suggest that inbreeding species are mute (Fig. 1B); however, most mute inbreeding species are clustered together into the same clade (Fig. 2), making the inheritance of the absence of sound production the most parsimonious explanation for these species. We would expect a loss of intersexual communication by sound production in species which inbreed and hence have reduced or no courtship behaviour; nonetheless, there is information on stridulatory organs or behaviour in the literature for only three of the eight known clades of inbreeding species (Fig. 2). Our results suggest that body size is the most important contributor to sound production, as size alone accurately predicts sound production in 70% of the species, in contrast to 71% when mating system and size are considered in the model (Methods, Fig. 3A,B).

**Figure 4:**
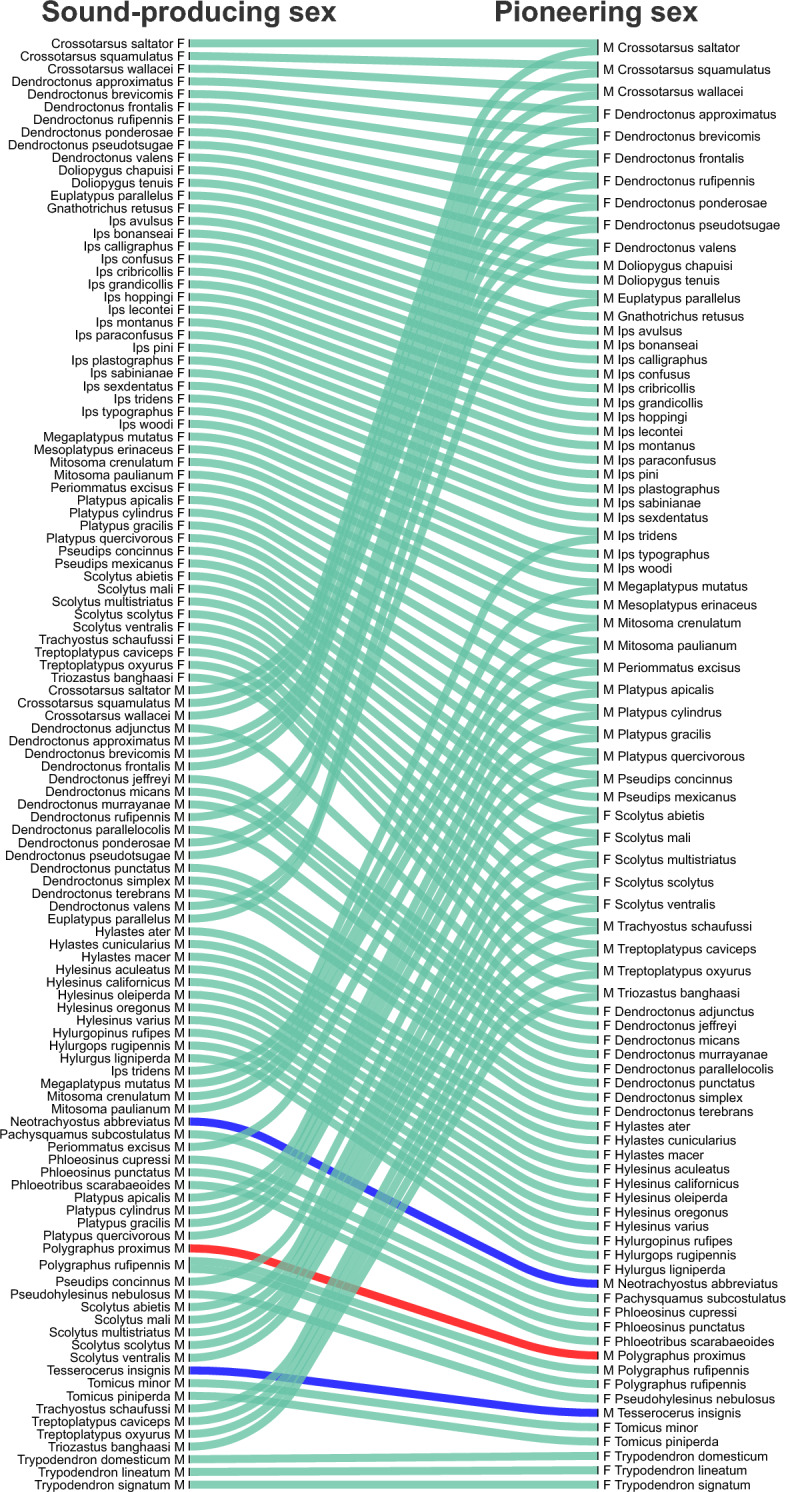
Acoustic communication always exists in the second-arriving sex. Sankey plot associating beetle sex (M: male, F: female) with acoustic signalling capabilities (sound-producing, left) with the beetle sex that arrives first to the host (pioneering, right). The sex opposite to the one that colonises the host for reproduction always possesses acoustic communication. *Neotrachyostus abbreviatus* (blue), and *Tesserocerus insignis* (blue) are noisy outliers since the author who reported the data (Menier, 1976) only had access to males. *Polygraphus proximus* (red) was the only true exception to the rule.

We also found that the sex opposite to the one that colonises the host for reproduction always possesses acoustic communication (pattern ii). This pattern holds for all species except *Polygraphus proximus* (Fig. 4). This is the only species where males are both the first-arriving sex and the only sex with sound production capabilities. Two other outliers also exist in the literature (*Neotrachyostus abbreviatus* and *Tesserocerus insignis*) (Fig. 4), but these can be explained by the fact that the author who reported that data (Menier, 1976; See Supplementary Table S1) only had access to males.

We suggest body size is the ultimate cause of the loss of acoustic signalling, but acknowledge that there must be other factors that limit communicating acoustically, as many of the losses that we report are in medium to large sized outbreeding species. Elytro-tergal stridulation is common in curculionids^8^, and its widespread occurrence in bark beetles and pinhole borers is almost certainly an ancestral trait within each subfamily^6^. In our dataset, we found 23 instances in which acoustic signalling has been lost (Fig. 2). In Platypodinae, the two mute genera (*Diapus* and *Genyocerus*) are closely related^15^ and represent one of the most specialised pinhole borer clades in both morphology and behaviour^16,17^. Stridulatory structures in these species may have been lost due to the extreme morphological adaptations in the elytra and pronotum, which they use to expel frass and in sexual selection contexts^16,17^, in a similar fashion to the loss of elytro-tergal stridulation in the genus *Ips* from Scolytinae^18^. All but one of the studied Corthylini species appear to be silent; *Gnathotrichus retusus* was reported by Barr (1969)^7^ to have a vertex-pronotal stridulatory organ, but we could find no other information on this species that could confirm its use of sound. Should this or other corthylines be found to stridulate, that would mean that sound production has been lost multiple times in this large clade. It is possible that corthylines represent just one loss of sound production. Xyleborini, the most speciose tribe in the Scolytinae, also represents just one origin of loss of acoustic behavior; all Xyleborini are haplodiploid inbreeding ambrosia beetles^9^. Multiple losses can be seen in the outbreeding bark beetle tribe Ipini, both in outbreeding harem polygynous species groups (two examples in *Ips* plus the genus *Orthotomicus*) and in the tridens group of pseudogamous^19^ species.

Bark beetles and pinhole borers occur in all regions of the world except Antarctica^20^. Studies on these beetles typically focus on species that attack and kill economically important trees, although this is known to occur in less than 1% of extant species^9^. Consequently, there is a strong bias in the literature towards tree-killing beetles of economically important species in temperate regions of the Northern Hemisphere^20^. This bias is also present in our dataset. Reporting sound production (or lack thereof) should be a priority, especially in the Platypodinae and the subtribes of the Scolytinae where acoustic information is unknown (e.g., Bothrosternini, Cactopinini, Hexacolini, Hyorrhinchini, Scolyplatipodini, Phrixosomini).

Our dataset reports on the three primary stridulatory mechanisms within bark beetles and pinhole borers: elytro-tergal, vertex-pronotal, and gula-prosternal stridulatory organs^7^. In pinhole borers (Platypodinae), only the elytro-abdominal stridulatory mechanism is known to occur^8,21^. However, new, or previously misinterpreted, structures have been identified recently as potential new types of stridulatory structures in several beetle families, such as elytro-tibial^22^, profemoral-mesocoxal^23^, and procoxal-mesocoxal^23^.

In this study, acoustic communication was characterized from the emitter standpoint, but body miniaturization could also have profound effects on hearing organs. As insects shrink in size, acoustic receptors such as tympanal membranes^24^ and chordonotal organs^25^ also decrease in size^26^. Consequently, smaller auditory structures might not be able to capture and amplify sound waves with the same efficiency as larger ones, thus reducing the ability of the beetle to perceive acoustic signals. On the other hand, the energetic and developmental costs associated with maintaining auditory systems^27^ might not be justifiable in miniaturized beetles, where vital functions are prioritised over sensory organs^28^. Acoustic communication, while beneficial, might become less essential compared to other survival functions, leading to its regression or complete loss, and ultimately impacting their communication strategies.

This is the first time a lower size bound for the presence of sound production has been established in any animal group. This study also stands as the sole rigorous investigation of the reasons why species stop signalling acoustically. This is also the first time that a general rule for the sex-dependence of acoustic signalling in beetles has been established. Our study sheds new light on the interplay between fundamental biological factors and the evolution of acoustic communication, fostering a deeper understanding of this phenomenon at lower size scales and of acoustic communication in general.

## Methods

We collected acoustic information for 55 bark beetle and pinhole borer species, including presence or absence of sound production, sex that stridulates, type of stridulatory organ, and audio recordings of the acoustic signals^6^. Our dataset was supplemented with a systematic comprehensive literature search performed in English, German, French, Italian, and Spanish. The literature search was performed on Scopus and Google Scholar using two keyword groups and their inter-group combinations: Group 1: bark beetle, ambrosia beetle, pinhole borer, Scolyti*, Platypodi*. Group 2: sound production, stridulation, acoustic communication: for example, “bark beetle AND sound production”. The search retrieved 704 documents, from which 93 were selected based on information in the abstract. Reports of stridulatory behaviours, plots of stridulatory signals, or descriptions of stridulatory organs were accepted as evidence of sound production in a species. The presence of stridulatory organs or sound production was reported for 194 species, but a significant number of these were synonyms, undescribed species, or misreported data, which reduced the number to 156 (Supplementary Table S1). Information about the sex that initiates gallery construction, mating system, feeding mode, phylogeny, and average body length of the species was taken mostly from book chapters, taxonomic monographs, and review papers (see Supplementary Table S1 for the full list of resources). Only complete cases were used in all the data analyses, i.e., we excluded species with missing values in the variables required to perform the respective analysis. Phylogenetic information (Supplementary Table S5) was obtained from published molecular phylogenies^15,29–33^ and used to generate the cladogram of the studied species (Fig. 2).

Reports of acoustic communication in species that had not been taxonomically described were not considered, as life-history and morphological information could not be found. Data from Sasakawa and Sasakawa (1981)^34^ and Sasakawa and Yoshiyasu (1983)^35^ have been reported as unreliable by several authors, and were thus disregarded from our analyses (see Lyal and King 1996^8^ and Bedoya 2020^18^ for detailed discussions).

2D multidimensional scaling (MDS) was selected as the ordination method for visualisation. MDS was performed on the measured morphological and life history variables 'size', 'mating system', and 'feeding mode'. The continuous variable 'size' was scaled [0–1] using Eq. (1) before performing the MDS. The categorical variables 'mating system' and 'feeding mode' were one-hot encoded using 'monogynous', 'phloeophagus', and 'female' (the most commonly occurring categories) as reference categories, respectively. The MDS was performed using the Python library scikit-learn, using the following parameters: 2 dimensions, 300 iterations, and Euclidean distance.

1 $$x_{norm} = \frac{x - \min \left( x \right)}{{\max \left( x \right) - {\text{min}}\left( x \right)}}$$

where $x_{norm}$ is the scaled variable, $x$ is the unscaled variable, and $\max \left( x \right)$ and ${\text{min}}\left( x \right)$ are the maximum and minimum values of $x$, respectively.

A binary logistic regression (Eq. (2)) was used to analyse the relationship between sound production, mating system and size. We accounted for pseudoreplication by considering each clade with mute species a single loss of sound production (Fig. 2). 'Presence of sound production' (*SP*) was used as a binary dependent variable. 'Mating system' was one-hot encoded using the most common category ('monogynous') as a reference category. The variable 'size' was scaled [0–1] using Eq. (1) before its use in the logistic regression. The results of the logistic regression were plotted on the MDS (Fig. 3A). Since there were only two output classes (i.e., presence or absence of sound production), a threshold of 0.5 was selected for the estimation of accuracy. The ground truth is presented for comparison in Fig. 3B.

2 $$SP = 1.716{ } + \left( {0.709} \right)S{ } - \left( {0.768} \right)HP{ } - \left( {0.416} \right)IP$$

where $SP$ is the dependent variable 'presence of sound production', and $S$, $HP$, and $IP$ are the independent variables 'size', 'harem polygyny', and 'inbreeding polygyny', respectively.

The swarm plot (Fig. 1B) was generated using the Python library seaborn. The Sankey plot (Fig. 4) was coded using the Python library floWeaver. The raw data and the code used to generate all the plots from these data are provided in Supplementary Tables S1, S3, S4, S5 and Supplementary Code S2. All algorithms were coded in Python 3.8.

### Supplementary Information

Supplementary Information 1.

Supplementary Information 2.

Supplementary Information 3.

Supplementary Information 4.

Supplementary Information 5.

## Data Availability

All the data and code required to replicate the results and figures of this manuscript are provided in the supplementary information.
