# Supplementary material for: Body size and sequence of host colonisation predict the presence of acoustic signalling in beetles
Source: Sci Rep. 2024 Jul 5;14:15532. doi: 10.1038/s41598-024-66108-8 (PMC11226610; doi:10.1038/s41598-024-66108-8)
Supplement: Supplementary file 1 — Supplementary Information 1. [file 41598_2024_66108_MOESM1_ESM.pdf]

```python
# -*- coding: utf-8 -*-
"""
Code for plotting species name vs size from the raw data.

"""
#%% Libraries
import pandas as pd
import matplotlib.pyplot as plt
import numpy as np

#%% Algorithm
### Read the CSV file
data = pd.read_csv("C:\\Users\CBA\Documents\Other\MetaBB\Table_python.csv", encoding =
'unicode_escape')
data['Sound'] = data['Sound'].replace("(Y)", "Y") # Replace "(Y)" with "Y" in the Sound column
data = data.dropna(subset=['Size']) # Remove rows with missing values

### Extract the desired columns
columns = ['Sound','Size','Genus','Species']
extracted_data = data[columns]

### Filter the data based on 'Sound' column
filtered_data = extracted_data[extracted_data['Sound'].isin(['Y', 'N'])] # Copy the dataframe,
but add an extra operation just in case there is a nan
filtered_data['Sound'] = filtered_data['Sound'].apply(lambda x: 1 if x == "Y" else 0) #
Transform Sound into binary

### Convert 'Size' column to numeric
filtered_data['Size'] = pd.to_numeric(filtered_data['Size'], errors='coerce')

### Sort the data by 'Size' column in descending order
sorted_data = filtered_data.sort_values('Size', ascending=False)

### Extract the required columns for the scatter plot
sizes = np.array(sorted_data['Size'])
species = np.array(sorted_data['Species'])
genus = np.array(sorted_data['Genus'])
names = [genus[j][0] + '. ' + name_spe for j, name_spe in enumerate(species)]
names = newxticklabels = [l if not i%2 else l+'-------------------------' for i,l in
enumerate(names)] #this is to offset some labels so that it can be easily read
sound_values = sorted_data['Sound']

### Create the scatter plot
fig, ax = plt.subplots(figsize=(20, 11))
```

```
plt.scatter(range(len(sizes)), sizes, c=sound_values, cmap='cividis', alpha=0.9, s=100,
edgecolors='black')
plt.xticks(range(len(names)), names, rotation=90, fontsize=18, fontstyle='italic',
fontname='Arial')
plt.gca().set(xlim=(-1, len(names)), ylim=(1,12.2))

### Set the axis labels
plt.xlabel('Species', fontsize=30,fontname='Arial')
plt.ylabel('Body size (mm)', fontsize=30,fontname='Arial')

### Add ticks and grid
ax.tick_params(axis='y', which='major', labelsize=20)
ax.yaxis.get_ticklocs(minor=True)

ax.xaxis.set_tick_params(which='minor', bottom=False)
ax.grid(linestyle='-', linewidth='0.1', color='grey')

### Add a dotted line at Size = 1.9
plt.axhline(y=1.9, color='black', linestyle='--', linewidth=2)
plt.axvline(x=139, color='black', linestyle='--', linewidth=2)

### Show the plot
#plt.show()
plt.tight_layout()

#%% Save figure
#fig.savefig(r"C:\Users\CBA\Documents\Other\MetaBB\Figures\Size_vs_Names_new.svg", bbox_inches
= "tight")
```

```python
# -*- coding: utf-8 -*-
"""
Code for plotting size vs mating system from the raw data.

"""
#%% Libraries
import pandas as pd
import matplotlib.pyplot as plt
import matplotlib.cm as cm
import seaborn as sns

#%% Algorithm

### Read the CSV file
data = pd.read_csv("C:\\Users\CBA\Documents\Other\MetaBB\Table_python.csv", encoding =
'unicode_escape')
data['Sound'] = data['Sound'].replace("(Y)", "Y") # Replace "(Y)" with "Y" in the Sound column
data = data.dropna(subset=['Size', 'Mating System']) # Remove rows with missing values

### Extract the desired columns
columns = ['Sound','Size','Mating System','Genus','Species']
extracted_data = data[columns]

### Filter the data based on 'Sound' column
filtered_data = extracted_data[extracted_data['Sound'].isin(['Y', 'N'])] # Copy the dataframe,
but add an extra operation just in case there is a nan
filtered_data['Sound'] = filtered_data['Sound'].apply(lambda x: 1 if x == "Y" else 0) #
Transform Sound into binary

### Convert 'Size' column to numeric
filtered_data['Size'] = pd.to_numeric(filtered_data['Size'], errors='coerce')

### Sort the data by 'Size' column in descending order
sorted_data = filtered_data.sort_values('Size', ascending=False)

### Create the scatter plot
fig, ax = plt.subplots(figsize=(10, 10))
sns.set_style("ticks")
cmap = cm.get_cmap('cividis')
g = sns.catplot(data=sorted_data, x="Mating System", y="Size", hue='Sound', kind='swarm', s =
7, linewidth=1, edgecolor='black', palette = {0: cmap(0), 1:cmap(256)}, legend = False,
height=6, aspect=0.8)

### Set the axis labels
plt.xlabel('Mating system', fontsize=20,fontname='Arial')
```

```
plt.ylabel('Body size (mm)', fontsize=20,fontname='Arial')

### Add ticks and grid
ax.tick_params(axis='y', which='major', labelsize=25)
ax.yaxis.get_ticklocs(minor=True)

ax.xaxis.set_tick_params(which='minor', bottom=False)
ax.grid(linestyle='-', linewidth='0.1', color='grey')

g.set_xticklabels(['Monogynous','Inbreeding','Polygynous'], fontsize=15)
g.set_yticklabels(fontsize=15)
g.set(ylim=(1,12.2))

# Add a dotted line at Size = 1.9
plt.axhline(y=1.9, color='black', linestyle='--', linewidth=2)

# Show the plot
plt.show()

#%% Save figure
#g.savefig(r"C:\Users\CBA\Documents\Other\MetaBB\Figures\Size_vs_MatSys_new.svg")
```

```python
# -*- coding: utf-8 -*-
"""
Code for plotting the logistic regression results on the 2D multi-dimimensional scaling from
the raw data.

"""

#%% Libraries
import pandas as pd
from sklearn.linear_model import LogisticRegression
import matplotlib.pyplot as plt
from sklearn.manifold import MDS
from sklearn.metrics import accuracy_score, precision_score, recall_score, f1_score,
roc_auc_score
from cmcrameri import cm

#%% Pre-processing
df = pd.read_csv("C:\\Users\\CBA\\Documents\\Other\\MetaBB\\S4_Table_python.csv", encoding =
'unicode_escape') #Read the spreadsheet
df['Sound'] = df['Sound'].replace("(Y)", "Y") # Replace "(Y)" with "Y" in the 'Sound' column
df = df.dropna(subset=['Size', 'Mating System']) # Remove rows with missing values
genus = df['Genus']
species = df['Species']
df = df[['Genus', 'Species', 'Size', 'Mating System', 'Sound', 'Feeding Mode']] # Select
columns of interest
df['Size'] = (df['Size'] - df['Size'].mean()) / df['Size'].std() #Scale the 'Size' column

#%% Create dummy variables
df = pd.concat([df, pd.get_dummies(df['Mating System'], prefix='Mat')], axis=1)
df = pd.concat([df, pd.get_dummies(df['Feeding Mode'], prefix='FMod')], axis=1)
df['Sound'] = df['Sound'].apply(lambda x: 1 if x == "Y" else 0) # Transform Sound into binary

#%% Remove original categorical variables
df = df.drop(['Mating System', 'Feeding Mode'], axis=1)

#%% copy the data frame for test
df_test = df.copy()

#%% Remove pseudoreplicates for training the logistic regression
df = df[df['Genus'] != 'Diapus'] # remove the genus Diapus
df = df[df['Species'] != 'vittatus'] # remove the Pteleobius vittatus
df = df[df['Species'] != 'fasciatum'] # remove the Monarthrum fasciatum
df = df[df['Genus'] != 'Pityophthorus'] # remove the genus Pityophthorus
df = df[df['Species'] != 'hampei'] # remove the species Hypothenemus hampei
df = df[df['Species'] != 'caelatus'] # remove the species Orthotomicus caelatus
df = df[df['Genus'] != 'Xyleborus'] # remove the genus Xyleborus
df = df[df['Genus'] != 'Xylosandrus'] # remove the genus Xylosandrus
df = df[df['Genus'] != 'Xyleborinus'] # remove the genus Xyleborinus
df = df[df['Genus'] != 'Cnestus'] # remove the genus Cnestus
df = df[df['Genus'] != 'Dryoxylon'] # remove the genus Dryoxylon
df = df[df['Genus'] != 'Ambrosiodmus'] # remove the genus Ambrosiodmus

#%% Logistic Regression

### Define X and y
X = df[['Size','Mat_HP','Mat_IP']]
y = df['Sound']

### Fit logistic regression model
model = LogisticRegression().fit(X, y)
```

```
### Print intercepts and coefficients
print('Intercept: ', model.intercept_)
print('Coefficients: ', model.coef_)

### Define X from test data
X_test = df_test[['Size','Mat_HP','Mat_IP']]

### Calculate predicted probabilities
probs = model.predict_proba(X_test)[:, 1]

#%% MDS Plot

### Perform MDS on the dummy variables
mds = MDS(n_components=2, random_state=6)
X_mds = mds.fit_transform(df_test.drop(['Genus','Species','Sound','Mat_M', 'FMod_PHL'],
axis=1))

### Create scatter plot of MDS
fig, ax = plt.subplots(figsize=(10, 10))
scatter = ax.scatter(X_mds[:,0], X_mds[:,1], c=probs, cmap=cm.batlow, s=200)

### Add colorbar
cbar = plt.colorbar(scatter)
cbar.ax.set_ylabel('Predicted probability of sound production',fontsize=25,fontname='Arial')
cbar.ax.tick_params(labelsize=18)

### Add axis labels
ax.set_xlabel('MDS : Dim 1',fontsize=25,fontname='Arial')
ax.set_ylabel('MDS : Dim 2',fontsize=25,fontname='Arial')
plt.xticks([])
plt.yticks([])

### Add text labels to each point
names = [genus[genus.index[j]][0] + '. ' + name_spe for j, name_spe in enumerate(species)]
text_values = names
for i, txt in enumerate(text_values):
    ax.text(X_mds[i,0], X_mds[i,1], txt, fontsize=6, fontstyle='italic', ha='center',
va='center')

### Plot
plt.show()

#%% Estimate Accuracy

### Threshold
probs[probs>=0.5]=1
probs[probs<0.5]=0

### Accuracy metrics
accuracy = accuracy_score(df_test['Sound'], probs) #Accuracy
precision = precision_score(df_test['Sound'], probs) #Precision
recall = recall_score(df_test['Sound'], probs) #Recall or Sensitivity
f1 = f1_score(df_test['Sound'], probs) #F1-Score

### Calculate AUC-ROC
probs = model.predict_proba(X_test)[:, 1]
auc = roc_auc_score(df_test['Sound'], probs)

### Print the metrics
print(f'Accuracy: {accuracy:.3f}')
print(f'Precision: {precision:.3f}')
```

```
print(f'Recall: {recall:.3f}')
print(f'F1-score: {f1:.3f}')
print(f'AUC-ROC: {auc:.3f}')

#%% Save figure
#fig.savefig(r"C:\Users\CBA\Documents\Other\MetaBB\Log_reg_fig.svg")
```

```python
# -*- coding: utf-8 -*-
"""
Code for plotting the ground truth on the 2D multi-dimimensional scaling from the raw data.

"""

#%% Libraries
import pandas as pd
import matplotlib.pyplot as plt
from sklearn.manifold import MDS
from cmcrameri import cm

#%% Pre-processing
df = pd.read_csv("C:\\Users\CBA\Documents\Other\MetaBB\Table_python.csv", encoding =
'unicode_escape') #Read the spreadsheet
df['Sound'] = df['Sound'].replace("(Y)", "Y") # Replace "(Y)" with "Y" in the 'Sound' column
df = df.dropna(subset=['Size', 'Mating System']) # Remove rows with missing values
genus = df['Genus']
species = df['Species']
df = df[['Size', 'Mating System', 'Sound', 'Feeding Mode']] # Select columns of interest
df['Size'] = (df['Size'] - df['Size'].mean()) / df['Size'].std() #Scale the 'Size' column

#%% Create dummy variables
df = pd.concat([df, pd.get_dummies(df['Mating System'], prefix='Mat')], axis=1)
df = pd.concat([df, pd.get_dummies(df['Feeding Mode'], prefix='FMod')], axis=1)
df['Sound'] = df['Sound'].apply(lambda x: 1 if x == "Y" else 0) # Transform Sound into binary

#%% Remove original categorical variables
df = df.drop(['Mating System', 'Feeding Mode'], axis=1)

#%% Ground truth
ground_truth = df['Sound']

#%% MDS Plot

### Perform MDS on the dummy variables
mds = MDS(n_components=2, random_state=6)
X_mds = mds.fit_transform(df.drop(['Sound','Mat_M', 'FMod_PHL'], axis=1))

### Create scatter plot of MDS
fig, ax = plt.subplots(figsize=(10, 10))
scatter = ax.scatter(X_mds[:,0], X_mds[:,1], c=ground_truth, cmap=cm.batlow, s=200)

### Add colorbar
#cbar = plt.colorbar(scatter)
```

```
#.ax.set_ylabel('Predicted probability of sound production',fontsize=25,fontname='Arial')
#cbar.ax.tick_params(labelsize=18)

### Add axis labels
ax.set_xlabel('MDS : Dim 1',fontsize=25,fontname='Arial')
ax.set_ylabel('MDS : Dim 2',fontsize=25,fontname='Arial')
plt.xticks([])
plt.yticks([])

### Add text labels to each point
names = [genus[genus.index[j]][0] + '. ' + name_spe for j, name_spe in enumerate(species)]
text_values = names
for i, txt in enumerate(text_values):
    ax.text(X_mds[i,0], X_mds[i,1], txt, fontsize=6, fontstyle='italic', ha='center',
va='center')

### Plot
plt.show()


#%% Save figure
#fig.savefig(r"C:\Users\CBA\Documents\Other\MetaBB\Figures\Ground_truth_new.svg")
```

```python
# -*- coding: utf-8 -*-
"""
Code for generating the Sankey plot.

Important! This code must be run in a Jupyter Notebook.

"""
import pandas as pd
import numpy as np
from floweaver import *

#%% Read the CSV file
data = pd.read_csv("C:\\Users\CBA\Documents\Other\MetaBB\Table_python.csv", encoding =
'unicode_escape')
data['Sound'] = data['Sound'].replace("(Y)", "Y") # Replace "(Y)" with "Y" in the Sound column
data = data.dropna(subset=['Sound', 'Sound Sex', 'Sex Initiating']) # Remove rows with missing
values
data = data[['Sound', 'Sound Sex', 'Sex Initiating', 'Genus', 'Species']] # Select columns of
interest
data = np.array(data)

#%% Data pre-processing
### Duplicate rows with 'B' in 'Sound Sex'
for index, row in reversed(list(enumerate(data))):
    print(index)
    if 'B' in data[index,1]:
        data = np.insert(data, index, data[index,:], axis=0)
        data[index,1] = 'M'
        data[index+1,1] = 'F'

### Duplicate rows with 'B' in 'Sex initiating'
for index, row in reversed(list(enumerate(data))):
    print(index)
    if 'B' in data[index,2]:
        data = np.insert(data, index, data[index,:], axis=0)
        data[index,2] = 'M'
        data[index+1,2] = 'F'

### group 'data' by Males and Females
data = sorted(data, key=lambda x: x[3])
data = sorted(data, key=lambda x: x[1])
data = np.array(data)

### Create 'source' and 'target' for the sankey plot combining information
```

```
source = data[:,3] + ' ' + data[:,4] + ' ' + data[:,1] # genus + species + sex that produces
sound
target = data[:,2] + ' ' + data[:,3] + ' ' + data[:,4] # sex that innitiates gallery
construction + genus + species

### sort target in alphabetical order (for visualisation)
target_sort = sorted(target) #this is optional, you have to enable it in the 'Sankey Plot'
section

dataf = pd.DataFrame({'Sound': data[:,0], 'Sound Sex': data[:,1], 'Sex Initiating': data[:,2],
'value': np.ones(len(data[:,0])), 'Genus': data[:,3], 'Species': data[:,4], 'source':source,
'target':target, 'target_sorted':target_sort })

#%% Sankey plot
nodes = {
    "start": ProcessGroup(list(dataf['source'])),
    "end": ProcessGroup(list(dataf['target'])),
}

ordering = [['start'], ['end']]
bundles = [Bundle('start', 'end')]

nodes['start'].partition = Partition.Simple('source', dataf['source'].unique())
nodes['end'].partition = Partition.Simple('target', dataf['target'].unique())

### Size options
size_options = dict(width=900, height=1700, margins=dict(left=210, right=310))

### Define Sankey plot
sdd = SankeyDefinition(nodes, bundles, ordering)

#%% Save image and plot
weave(sdd,dataf,palette='Set2_8').to_widget(**size_options) #just plot
#weave(sdd,dataf,palette='Set2_8').to_widget(**size_options).auto_save_svg('C:\\Users\CBA\Docu
ments\Other\MetaBB\Figures\Sankey_new_test.svg') #plot and save
```

```python
# -*- coding: utf-8 -*-
"""
Code for generating the cladogram from the distance matrix.
"""

#%% Libraries
import numpy as np
import scipy.cluster.hierarchy as sch
import matplotlib.pyplot as plt
import pandas as pd

#%% Read the CSV file with the distance matrix

### Read data
data = pd.read_csv("C:\\Users\\CBA\\Documents\\Other\\MetaBB\\S5_Table_Distance_Matrix.csv",
encoding = 'unicode_escape')

### Transform the data into a np array
data = data.to_numpy()
species_names = data[:,1]
distance_matrix = data[:,2:]

### Convert the distance matrix to a condensed distance matrix
condensed_distance_matrix = sch.distance.squareform(distance_matrix)

#%% Perform hierarchical/agglomerative clustering
linkage_matrix = sch.linkage(condensed_distance_matrix, method='average')

#%% Plot the dendrogram
plt.figure(figsize=(10, 5))
dendrogram = sch.dendrogram(linkage_matrix, labels=species_names, color_threshold=0.7,
leaf_font_size=4, link_color_func=lambda x: 'black')

### remove the boxing around the dendrogram
plt.gca().spines['right'].set_visible(False)
plt.gca().spines['left'].set_visible(False)
plt.gca().spines['bottom'].set_visible(False)
plt.gca().spines['top'].set_visible(False)

#remove y ticks
plt.yticks([])

#%% Save the dendrogram to a .svg file
plt.tight_layout()
plt.savefig("C:\\Users\\CBA\\Documents\\Other\\MetaBB\\cladogram.svg")
plt.show()
```
