# Supplementary material for: Body size and sequence of host colonisation predict the presence of acoustic signalling in beetles
Source: Sci Rep. 2024 Jul 5;14:15532. doi: 10.1038/s41598-024-66108-8 (PMC11226610; doi:10.1038/s41598-024-66108-8)
Supplement: Supplementary file 2 — Supplementary Information 2. [file 41598_2024_66108_MOESM2_ESM.pdf]

S1 Table. Literature review of bark beetle and pinhole borer sizes and life history data. **Sound** (species with acoustic communication, **Y**: yes, and acoustic signals have been reported, (**Y)**: yes, but only stridulatory organs or stridulatory behaviour reported, **N**: no). **Sex prod. Sound** (beetle sex with sound production capabilities, **M**: male, **F**: female, **B**: both). **Organ** (type of stridulatory organ, **ET**: elytro-tergal, **VP**: vertex-pronotal, **GP**: gula-prosternal). **Mating System**: type of mating system (**IB**: inbreeding polygynous, **M**: monogynous, **HP**: harem polygynous). **Sex Initiating** (**M**: males arrive at host plant first and initiate gallery construction, **F**: females arrive at host plant first and initiate gallery construction, **B**: both sexes can initiate gallery construction). **Feeding mode** (**PHL:** phloeophagy, **XYL:** xylomycetophagy, **XPH:** xylophagy, **MYE:** myelophagy, **SPE:** spermatophagy). **Size** (average body size for the species, in mm). **Sound Ref**: References used to obtain information about sound production. **Size Ref:** References used to obtain body size. **Life History Ref:** References used for mating system, feeding mode, and the sex that arrives first to the host.
***author reported information for the whole genus.

#	Subfamily	Tribe	Genus	Species	Sound	Sex prod. Sound	Organ	Mating System	Sex Initiating	Feeding Mode	Size	Sound Ref.	Size Ref.	Life History Ref.	Notes
1	Platypodinae	Platypodini	*Crossotarsus*	*saltator*	(Y)	B	ET	M	M	XYL	3.2	Menier, 1976	Schedl, 1935	Kirkendall et al., 2015*	Original description. Holotype
2	Platypodinae	Platypodini	*Crossotarsus*	*squamulatus*	(Y)	B	ET	M	M	XYL	5.5	Menier, 1976	Chapuis, 1865	Kirkendall et al., 2015*	
3	Platypodinae	Platypodini	*Crossotarsus*	*wallacei*	(Y)	B	ET	M	M	XYL	12.0	Menier, 1976	Chapuis, 1865	Kirkendall et al., 2015*	
4	Platypodinae	Platypodini	*Doliopygus*	*chapuisi*	(Y)	F	ET	M	M	XYL	7.0	Menier, 1976	Duvivier, 1891	Kirkendall et al., 2015*	Original description. Holotype
5	Platypodinae	Platypodini	*Doliopygus*	*tenuis*	(Y)	F	ET	M	M	XYL	2.8	Menier, 1976	Atkinson, 2021	Kirkendall et al., 2015*	
6	Platypodinae	Platypodini	*Euplatypus*	*parallelus*	Y	B	ET	M	M	XYL	4.2	Bedoya et al., 2021	Maruthadurai et al., 2014	Kirkendall et al., 2015*	Synonym with *Platypus linearis*
7	Platypodinae	Platypodini	*Megaplatypus*	*mutatus*	(Y)	B		M	M	XYL	8.0	Ceriani-Nakamurakare et al., 2022	Ceriani-Nakamurakare et al., 2022	Ceriani-Nakamurakare et al., 2022	Only the presence of stridulatory signals was reported
8	Platypodinae	Platypodini	*Mesoplatypus*	*erinaceus*	(Y)	F	ET	M	M	XYL		Menier, 1976		Kirkendall et al., 2015*	Only females were examined (Menier, 1976)
9	Platypodinae	Platypodini	*Neotrachyostus*	*abbreviatus*	(Y)	M	ET	M	M	XYL	7.3	Menier, 1976	Chapuis, 1865	Kirkendall et al., 2015*	Only males were examined (Menier, 1976)
10	Platypodinae	Platypodini	*Platypus*	*apicalis*	Y	B	ET	M	M	XYL	6.8	Bedoya et al., 2021	Zervos, 1980	Kirkendall et al., 2015*	
11	Platypodinae	Platypodini	*Platypus*	*cylindrus*	(Y)	B	ET	M	M	XYL	4.8	Menier, 1976	Chapuis, 1865	Kirkendall et al., 2015*	
12	Platypodinae	Platypodini	*Platypus*	*gracilis*	Y	B	ET	M	M	XYL	5.0	Bedoya et al., 2021	Zervos, 1980	Kirkendall et al., 2015*	
13	Platypodinae	Platypodini	*Platypus*	*quercivorous*	Y	B	ET	M	M	XYL		Ohya and Kinuura, 2001; Kobayashi and Ueda, 2002		Kirkendall et al., 2015*	
14	Platypodinae	Platypodini	*Trachyostus*	*schaufussi*	(Y)	B	ET	M	M	XYL		Menier, 1976		Kirkendall et al., 2015*	
15	Platypodinae	Platypodini	*Treptoplatypus*	*caviceps*	Y	B	ET	M	M	XYL	6.8	Ytsma 1988; Bedoya et al., 2021	Zervos, 1980	Kirkendall et al., 2015*	
16	Platypodinae	Platypodini	*Treptoplatypus*	*oxyurus*	(Y)	B	ET	M	M	XYL	4.8	Menier, 1976; Žarković et al., 2022	Žarković et al., 2022	Kirkendall et al., 2015*	Synonym with *Platypus oxyurus*

17	Platypodinae	Platypodini	*Triozastus*	*banghaasi*	(Y)	B	ET	M	M	XYL		Menier, 1976		Kirkendall et al., 2015*	
18	Platypodinae	Tesserocerini	*Diapus*	*quinquespinatus*	N			M	M	XYL	2.5	Menier, 1976	Chapuis, 1865	Kirkendall et al., 2015*	
19	Platypodinae	Tesserocerini	*Genyocerus*	*biporus*	N			M	M	XYL	4.3	Menier, 1976	Beaver and Liu, 2007	Kirkendall et al., 2015*	Synonym with *Diacavus biporus*
20	Platypodinae	Tesserocerini	*Mitosoma*	*crenulatum*	(Y)	B	ET	M	M	XYL	3.5	Menier, 1976	Chapuis, 1865	Kirkendall et al., 2015*	
21	Platypodinae	Tesserocerini	*Mitosoma*	*paulianum*	(Y)	B	ET	M	M	XYL		Menier, 1976		Kirkendall et al., 2015*	
22	Platypodinae	Tesserocerini	*Periommatus*	*excisus*	(Y)	B	ET	M	M	XYL		Menier, 1976		Kirkendall et al., 2015*	
23	Platypodinae	Tesserocerini	*Tesserocerus*	*insignis*	(Y)	M	ET	M	M	XYL	7.5	Menier, 1976	Chapuis, 1865	Kirkendall et al., 2015*	Only males were examined (Menier, 1976)
24	Scolytinae	Corthylini	*Gnathotrichus*	*deleoni*	N			M	M	XYL	3.1	Bedoya et al., 2021	Wood, 1982	Wood, 1982*	
25	Scolytinae	Corthylini	*Gnathotrichus*	*materiarius*	N			M	M	XYL	2.4	Bedoya et al., 2021	Wood, 1982	Batra, 1963; Kirkendall, 1983*; Witkowski et al., 2022	
26	Scolytinae	Corthylini	*Gnathotrichus*	*retusus*	(Y)	F	VP	M	M	XYL	3.5	Barr, 1969	Wood, 1982	Kirkendall, 1983*; Liu and McLean, 1993	
27	Scolytinae	Corthylini	*Gnathotrichus*	*sulcatus*	N			M	M	XYL	3.2	Bedoya et al., 2021	Wood, 1982	Kirkendall, 1983	
28	Scolytinae	Corthylini	*Monarthrum*	*fasciatum*	N			HP	M	XYL	2.6	Bedoya et al., 2021	Wood, 1982	Kirkendall, 1983	
29	Scolytinae	Corthylini	*Monarthrum*	*mali*	N			HP	M	XYL	2.1	Bedoya et al., 2021	Wood, 1982	Kirkendall, 1983*	
30	Scolytinae	Corthylini	*Pityophthorus*	*annectens*	N			HP	M	PHL	1.5	Bedoya et al., 2021	Wood, 1982	Wood, 1982*	
31	Scolytinae	Corthylini	*Pityophthorus*	*concentralis*	N			HP	M	PHL	1.4	Bedoya et al., 2021	Wood, 1982	Wood, 1982*	
32	Scolytinae	Corthylini	*Pityophthorus*	*confusus*	N			HP	M	PHL	2.2	Bedoya et al., 2021	Wood, 1982	Wood, 1982*; Kirkendall 1983*; Deyrup and Atkinson, 1987	
33	Scolytinae	Corthylini	*Pityophthorus*	*consimilis*	N			HP	M	PHL	1.5	Bedoya et al., 2021	Wood, 1982	Wood, 1982*	
34	Scolytinae	Corthylini	*Pityophthorus*	*liquidambarus*	N			HP	M	PHL	1.4	Bedoya et al., 2021	Wood, 1982	Wood, 1982*	
35	Scolytinae	Corthylini	*Pityophthorus*	*pulicarius*	N			HP	M	MYE	1.7	Bedoya et al., 2021	Wood, 1982	Bright, 1981; Deyrup and Atkinson, 1987	
36	Scolytinae	Corthylini	*Pseudopityophthorus*	*minutissimus*	N			M	M	PHL	1.7	Bedoya et al., 2021	Wood, 1982	McMullen, 1955; Deyrup and Atkinson, 1987	
37	Scolytinae	Trypophloeini	*Hypothenemus*	*eruditus*	N			IP	F	PHL	1.2	Bedoya et al., 2021	Wood, 1982	Wood, 1982; Kirkendall 1983; Deyrup and Atkinson, 1987	
38	Scolytinae	Trypophloeini	*Hypothenemus*	*hampei*	N			IP	F	SPE	1.6	Bedoya et al., 2021	Wood, 1982	Wood, 1982; Kirkendall 1983; Deyrup and Atkinson, 1987	
39	Scolytinae	Dryocoetini	*Dactylotrypes*	*longicollis*	N			M	F	SPE	2.0	Bedoya et al., 2021	LaBonte and Takahashi, 2012	LaBonte and Takahashi, 2012, Kirkendall et al., 2015*	
40	Scolytinae	Hylastini	*Hylastes*	*angustatus*	(Y)		ET	M	F	PHL	2.9	Marcu, 1931	Balachowsky, 1949	Balachowsky, 1949; Wood, 1982*; Kirkendall et al., 2015*; Munro 1917*	

41	Scolytinae	Hylastini	*Hylastes*	*ater*	Y	M	ET	M	F	PHL	4.5	Bedoya et al., 2021	Milligan, 1978	Reay, 2000	
42	Scolytinae	Hylastini	*Hylastes*	*attenuatus*	(Y)		ET	M	F	PHL	2.3	Marcu, 1931	Balachowsky, 1949	Balachowsky, 1949; Wood, 1982*; Kirkendall et al., 2015*; Munro 1917*	
43	Scolytinae	Hylastini	*Hylastes*	*brunneus*	(Y)		ET	M	F	PHL	3.7	Marcu, 1931	Pfeffer, 1955	Wood, 1982*; Kirkendall et al., 2015*; Munro 1917*	
44	Scolytinae	Hylastini	*Hylastes*	*cunicularius*	(Y)	M	ET	M	F	PHL	3.9	Marcu, 1931; Wichmann, 1912	Balachowsky, 1949	Wood, 1982*; Kirkendall et al., 2015*; Munro 1917*	
45	Scolytinae	Hylastini	*Hylastes*	*macer*	(Y)	M	ET	M	F	PHL	5.6	Barr, 1969	Wood, 1982	Wood, 1982*; Kirkendall et al., 2015*; Munro 1917*	
46	Scolytinae	Hylastini	*Hylastes*	*opacus*	(Y)		ET	M	F	PHL	3.0	Marcu, 1931	Balachowsky, 1949	Balachowsky, 1949; Wood, 1982*; Kirkendall et al., 2015*; Munro 1917*	
47	Scolytinae	Hylastini	*Hylastes*	*porculus*	N			M	F	PHL	4.4	Bedoya et al., 2021	Wood, 1982	Wood, 1982	
48	Scolytinae	Hylastini	*Hylurgops*	*glabratus*	(Y)		ET	M		PHL	4.8	Wichmann, 1912; Marcu, 1931	Balachowsky, 1949	Balachowsky, 1949; Wood, 1982*; Kirkendall et al., 2015*	Synonym with *Hylurgops decumanus*
49	Scolytinae	Hylastini	*Hylurgops*	*interstitialis*	(Y)	M	ET	M		PHL	4.7	Lyal and King, 1996	Park, 2016	Kirkendall et al., 2015*	
50	Scolytinae	Hylastini	*Hylurgops*	*palliatus*	(Y)		ET	M		PHL	2.9	Wichmann, 1912; Munro, 1917; Marcu, 1931	Balachowsky, 1949	Balachowsky, 1949; Kirkendall et al., 2015*	Synonym with *Hylurgops palliatus* and *Hylastes palliatus*
51	Scolytinae	Hylastini	*Hylurgops*	*rugipennis*	Y	M	ET	M	F	PHL	4.4	Barr, 1969; Oester et al., 1978	Wood, 1982	Oester et al., 1978	
52	Scolytinae	Hylastini	*Pachysquamus*	*subcostulatus*	Y	M	ET	M	F	PHL	4.0	Bedoya et al., 2021	Mercado-Vélez and Negrón, 2014	Wood, 1982	Sound and life history taken from *Hylurgops subcosutlatus* (synonym)
53	Scolytinae	Hylesinini	*Hylastinus*	*fankhauseri*	(Y)		ET	M		PHL	2.4	Marcu, 1931	Pfeffer, 1984	Balachowsky, 1949; Kirkendall et al., 2015*	
54	Scolytinae	Hylesinini	*Hylesinus*	*aculeatus*	Y	M	ET	M	F	PHL	2.5	Bedoya et al., 2019b	Wood, 1982	Wood, 1982; Kirkendall 1983; Deyrup and Atkinson 1987	
55	Scolytinae	Hylesinini	*Hylesinus*	*californicus*	Y	M	ET	M	F	PHL	2.6	Vernoff and Rudinsky, 1980	Wood, 1982	Langor and Hergert, 1993	
56	Scolytinae	Hylesinini	*Hylesinus*	*crenatus*	N			M		PHL	4.8	Wichmann, 1912; Kleine, 1921; Marcu, 1930d	Petrov, 2018	Wood, 1982*; Kirkendall et al., 2015*	Kleine says that there is presence of stridulatory organs, but no stridulatory behaviour
57	Scolytinae	Hylesinini	*Hylesinus*	*oleiperda*	Y	M	ET	M	F	PHL	3.0	Rudinsky and Vallo, 1978	Balachowsky, 1949	Balachowsky, 1949; Kirkendall 1983	Synonym with *Hylesinus toranio*
58	Scolytinae	Hylesinini	*Hylesinus*	*oregonus*	Y	M	ET	M	F	PHL	2.9	Vernoff and Rudinsky, 1980	Wood, 1982	Wood, 1982; Kirkendall et al., 2015*; Vernoff and Rudinsky, 1980	Synonym with *Leperisinus oregonus*
59	Scolytinae	Hylesinini	*Hylesinus*	*orni*	(Y)		ET	M		PHL	2.7	Marcu, 1930d	Balachowsky, 1949	Balachowsky, 1949; Wood, 1982*; Kirkendall et al., 2015*	Synonym with *Leperisinus orni*
60	Scolytinae	Hylesinini	*Hylesinus*	*varius*	Y	M	ET	M	F	PHL	2.9	Rudinsky and Vallo, 1978	Balachowsky, 1949	Løyning and Kirkendall, 1999;	Synonym with *Leperisinus fraxini*

														Rudinsky and Vallo, 1978	
61	Scolytinae	Hylesinini	*Pteleobius*	*kraatzi*	N			M		PHL	2.0	Kleine, 1921; Marcu, 1930d	Balachowsky, 1949	Balachowsky, 1949; Kirkendall et al., 2015*	
62	Scolytinae	Hylesinini	*Pteleobius*	*vittatus*	N			M		PHL	2.0	Kleine, 1921; Marcu, 1930d	Balachowsky, 1949	Balachowsky, 1949; Kirkendall et al., 2015*	
63	Scolytinae	Hylurgini	*Dendroctonus*	*adjunctus*	Y	M	ET	M	F	PHL	4.3	Bedoya et al., 2021	Wood, 1982	Wood, 1982	Synonym with *Dendroctonus convexifrons*
64	Scolytinae	Hylurgini	*Dendroctonus*	*approximatus*	Y	B	ET	M	F	PHL	5.6	Yturralde and Hofstetter, 2015	Wood, 1982	Wood, 1982	
65	Scolytinae	Hylurgini	*Dendroctonus*	*brevicomis*	Y	B	ET	M	F	PHL	3.5	Bedoya et al., 2021	Wood, 1982	Wood, 1982	
66	Scolytinae	Hylurgini	*Dendroctonus*	*frontalis*	Y	B	ET	M	F	PHL	2.8	Bedoya et al., 2021	Wood, 1982	Wood, 1982	
67	Scolytinae	Hylurgini	*Dendroctonus*	*jeffreyi*	(Y)	M	ET	M	F	PHL	5.7	Wood, 1963; Lyon, 1958	Wood, 1982	Wood, 1982	
68	Scolytinae	Hylurgini	*Dendroctonus*	*micans*	(Y)	M	ET	IP	F	PHL	7.5	Wood, 1963	Balachowsky, 1949	Balachowsky, 1949	
69	Scolytinae	Hylurgini	*Dendroctonus*	*murrayanae*	(Y)	M	ET	M	F	PHL	6.6	Hopkins, 1909; Wood, 1963	Wood, 1982	Wood, 1982	
70	Scolytinae	Hylurgini	*Dendroctonus*	*rufipennis*	Y	B	ET	M	F	PHL	5.7	Lyon, 1958; Rudinsky and Michael, 1973; Keeling, 2021	Wood, 1982	Wood, 1982*	Synonym with *Dendroctonus engelmanni* and *D. obesus*
71	Scolytinae	Hylurgini	*Dendroctonus*	*parallelocolis*	(Y)	M	ET	M	F	PHL	5.7	Wood, 1963	Wood, 1982	Wood, 1982*	Synonym with *Dendroctonus aztecus*
72	Scolytinae	Hylurgini	*Dendroctonus*	*ponderosae*	Y	B	ET	M	F	PHL	5.2	Yandell, 1984; Rudinsky and Michael, 1973; Fleming et al., 2013	Wood, 1982	Wood, 1982	Synonym with *Dendroctonus monticolae*
73	Scolytinae	Hylurgini	*Dendroctonus*	*pseudotsugae*	Y	B	ET	M	F	PHL	5.7	Bedoya et al., 2021; Rudinsky and Michael, 1973	Wood, 1982	Wood, 1982	
74	Scolytinae	Hylurgini	*Dendroctonus*	*punctatus*	(Y)	M	ET	IP	F	PHL	6.1	Wood, 1963	Wood, 1982	Wood, 1982	
75	Scolytinae	Hylurgini	*Dendroctonus*	*simplex*	(Y)	M	ET	M	F	PHL	4.2	Wood, 1963	Wood, 1982	Wood, 1982	
76	Scolytinae	Hylurgini	*Dendroctonus*	*terebrans*	Y	M	ET	M	F	PHL	6.3	Bedoya et al., 2021	Wood, 1982	Wood, 1982	
77	Scolytinae	Hylurgini	*Dendroctonus*	*valens*	Y	B	ET	M	F	PHL	6.8	Rudinsky and Michael, 1973; Ryker, 1988; Lindeman and Yack, 2015	Wood, 1982	Wood, 1982	
78	Scolytinae	Hylurgini	*Hylurgopinus*	*rufipes*	Y	M	ET	M	F	PHL	2.4	Swedenborg et al., 1989	Wood, 1982	Wood, 1982; Swedenborg et al., 1988	
79	Scolytinae	Hylurgini	*Hylurgus*	*ligniperda*	Y	M	ET	M	F	PHL	4.9	Bedoya et al., 2019a	Park, 2016	Fabre and Carle, 1975; Bain, 1977	
80	Scolytinae	Hylurgini	*Pachycotes*	*peregrinus*	N			M	F	XPH	4.7	Bedoya et al., 2021	Bain, 1977	Bain, 1977	
81	Scolytinae	Hylurgini	*Pseudohylesinus*	*nebulosus*	Y	M	ET	M	F	PHL	2.6	Oester et al., 1981	Wood, 1982	Wood, 1982	
82	Scolytinae	Hylurgini	*Tomicus*	*minor*	(Y)	M	ET	M	F	PHL	4.2	Marcu, 1931; Wichmann, 1912	Kirkendall, 2008	Kirkendall, 2008*	Synonym with *Myelophilus minor*
83	Scolytinae	Hylurgini	*Tomicus*	*piniperda*	(Y)	M	ET	M	F	PHL	4.4	Barr, 1969	Kirkendall, 2008	Kirkendall, 2008*	

84	Scolytinae	Hylurgini	*Xylechinus*	*pilosus*	(Y)		ET	M		XPH	2.4	Wichmann, 1912	Grüne, 1979	Balachowsky, 1949; Moucheron, 2016; Gninenko 2020	
85	Scolytinae	Hylurgini	*Chaetoptelius*	*vestitus*	(Y)		ET	M	F	PHL	2.9	Whichmann, 1912	Balachowsky, 1949	Balachowsky, 1949; Kirkendall et al., 2015*	Synonym with *Pteleobius vestitus*
86	Scolytinae	Ipini	*Ips*	*avulsus*	Y	F	VP	HP	M	PHL	2.5	Bedoya et al., 2021	Wood, 1982	Wood, 1982	
87	Scolytinae	Ipini	*Ips*	*bonanseai*	(Y)	F	VP	HP	M	PHL	3.2	Barr, 1969	Wood, 1982	Wood, 1982	
88	Scolytinae	Ipini	*Ips*	*borealis*	N			HP	M	PHL	3.2	Barr, 1969	Wood, 1982	Wood, 1982	
89	Scolytinae	Ipini	*Ips*	*calligraphus*	Y	F	VP	HP	M	PHL	4.9	Bedoya et al., 2021	Wood, 1982	Wood, 1982	
90	Scolytinae	Ipini	*Ips*	*confusus*	Y	F	VP	HP	M	PHL	3.9	Lukic et al., 2021; Lewis and Cane, 1992	Wood, 1982	Wood, 1982	Synonym with *Ips paraconfusus*
91	Scolytinae	Ipini	*Ips*	*cribricollis*	(Y)	F	VP	HP	M	PHL	3.8	Barr, 1969	Wood, 1982	Wood, 1982	Size taken from *Ips grandicollis* (Synonym)
92	Scolytinae	Ipini	*Ips*	*emarginatus*	N			HP	M	PHL	6.2	Barr, 1969	Wood, 1982	Wood, 1982	
93	Scolytinae	Ipini	*Ips*	*grandicollis*	Y	F	VP	HP	M	PHL	3.8	Bedoya et al., 2021	Wood, 1982	Wood, 1982	
94	Scolytinae	Ipini	*Ips*	*hoppingi*	Y	F		HP	M	PHL	3.9	Lewis and Cane, 1992	Wood, 1982	Wood, 1982	
95	Scolytinae	Ipini	*Ips*	*hunteri*	N			HP	M	PHL	3.7	Barr, 1969	Wood, 1982	Wood, 1982	
96	Scolytinae	Ipini	*Ips*	*knausi*	N			HP	M	PHL	5.7	Barr, 1969	Wood, 1982	Wood, 1982	
97	Scolytinae	Ipini	*Ips*	*lecontei*	Y	F	VP	HP	M	PHL	4.4	Barr, 1969; Lewis and Cane, 1992	Wood, 1982	Wood, 1982	
98	Scolytinae	Ipini	*Ips*	*montanus*	(Y)	F	VP	HP	M	PHL	5.0	Barr, 1969	Wood, 1982	Wood, 1982	
99	Scolytinae	Ipini	*Ips*	*paraconfusus*	Y	F	VP	HP	M	PHL	3.9	Barr, 1969; Lewis and Cane, 1992	Wood, 1982	Wood, 1982	
100	Scolytinae	Ipini	*Ips*	*perroti*	N			HP	M	PHL	3.1	Barr, 1969	Wood, 1982	Wood, 1982	
101	Scolytinae	Ipini	*Ips*	*perturbatus*	N			HP	M	PHL	4.4	Barr, 1969	Wood, 1982	Wood, 1982	
102	Scolytinae	Ipini	*Ips*	*pilifrons*	N			HP	M	PHL	4.7	Barr, 1969	Wood, 1982	Wood, 1982	Size taken from *Ips pilifrons pilifrons* (Synonym). Also Synonym with *Ips utahensis* and *Ips pilifrons utahensis*
103	Scolytinae	Ipini	*Ips*	*pini*	Y	F	VP	HP	M	PHL	3.8	Sivalinghem 2011; Bedoya et al., 2021	Wood, 1982	Wood, 1982	Oester and Rudinsky (1975) report sounds for males; however, no stridulatory organ has ever been found
104	Scolytinae	Ipini	*Ips*	*plastographus*	(Y)	F	VP	HP	M	PHL	4.6	Barr, 1969	Wood, 1982	Wood, 1982	
105	Scolytinae	Ipini	*Ips*	*sabinianae*	(Y)	F	VP	HP	M	PHL	3.0	Barr, 1969	Wood, 1982	Wood, 1982	Synonym with *Orthotomicus sabinianae* and *Ips spinifer*. Size taken from *Ips spinifer* (synonym).
106	Scolytinae	Ipini	*Ips*	*sexdentatus*	(Y)	F	VP	HP	M	PHL	7.4	Nunberg, 1950; Michalski, 1925	Balachowsky, 1949	Wood, 1982	
107	Scolytinae	Ipini	*Ips*	*tridens*	Y	B		HP	M	PHL	4.1	Oester and Rudinsky, 1975	Wood, 1982	Wood, 1982	Size taken from *Ips tridens tridens* (Synonym). Barr

															(1969) says that there is no sound production.
108	Scolytinae	Ipini	*Ips*	*typographus*	Y	F	GP	HP	M	PHL	5.5	Rudinsky, 1979	Balachowsky, 1949	Wood, 1982	
109	Scolytinae	Ipini	*Ips*	*woodi*	(Y)	F	VP	HP	M	PHL	4.1	Barr, 1969	Wood, 1982	Wood, 1982	
110	Scolytinae	Ipini	*Orthotomicus*	*caelatus*	N			HP	M	PHL	2.9	Bedoya et al., 2021	Wood, 1982	Wood, 1982*; Phillips et al., 1989	
111	Scolytinae	Ipini	*Orthotomicus*	*latidens*	N			HP	M	PHL	3.0	Barr, 1969	Wood, 1982	Wood, 1982	Synonym with *Ips latidens*
112	Scolytinae	Ipini	*Pseudips*	*concinnus*	Y	B	GP	HP	M	PHL	4.1	Barr, 1969; Oester and Rudinsky, 1975	Wood, 1982	Camacho-Pantoja, 2012	Synonym with *Ips concinnus*
113	Scolytinae	Ipini	*Pseudips*	*mexicanus*	(Y)	F	GP	HP	M	PHL	4.3	Barr, 1969	Wood, 1982	Smith et al., 2009	Synonym with *Ips mexicanus*
114	Scolytinae	Phloeosinini	*Dendrosinus*	*bourreriae*	(Y)			M		XPH	3.9	Schwarz, 1920	Wood, 1982	Schwarz, 1920; Wood, 1982	
115	Scolytinae	Phloeosinini	*Phloeosinus*	*aubei*	(Y)		ET	HP	M	PHL	2.4	Zocchi, 1956 (in Lyal and King, 1996)	Balachowsky, 1949	Balachowsky, 1949; Kirkendall et al., 2015*; Belhabib et al, 2009	Synonym with *Phloeosinus bicolor*
116	Scolytinae	Phloeosinini	*Phloeosinus*	*cupressi*	Y	M	ET	M	F	PHL	3.1	Bedoya et al., 2021	Wood, 1982	Wood, 1982	
117	Scolytinae	Phloeosinini	*Phloeosinus*	*dentatus*	N			M	F	PHL	2.3	Bedoya et al., 2021	Wood, 1982	Wood, 1982	
118	Scolytinae	Phloeosinini	*Phloeosinus*	*punctatus*	(Y)	M	ET	M	F	PHL	2.7	Barr, 1969	Wood, 1982	Wood, 1982	
119	Scolytinae	Phloeotribini	*Phloeotribus*	*caucasicus*	(Y)		ET	M		PHL	1.9	Marcu, 1931	Schott and Callot, 1994	Schott and Callot, 1994; Kirkendall et al., 2015*	
120	Scolytinae	Phloeotribini	*Phloeotribus*	*liminaris*	N			M	F	PHL	2.1	Bedoya et al., 2021	Wood, 1982	Wood, 1982	
121	Scolytinae	Phloeotribini	*Phloeotribus*	*rhododactylus*	(Y)		ET			PHL	2.3	Wichmann, 1912	Lindemann, 1875	Lindemann, 1875; Fiala and Holuša, 2020	Synonym with *Phloeophthorus rhododactylus*
122	Scolytinae	Phloeotribini	*Phloeotribus*	*scarabaeoides*	(Y)	M	ET	M	F	PHL	2.2	Russo, 1938	Balachowsky, 1949	Balachowsky, 1949	
123	Scolytinae	Phloeotribini	*Phloeotribus*	*spinulosus*	(Y)		ET			PHL	2.0	Wichmann, 1912	Balachowsky, 1949	Balachowsky, 1949; Kirkendall et al., 2015*	Synonym with *Phthorophloeus spinulosus*
124	Scolytinae	Polygraphini	*Carphoborus*	*bicornis*	N			HP	M	PHL	1.5	Bedoya et al., 2021	Wood, 1986	Wood, 1982*	
125	Scolytinae	Polygraphini	*Polygraphus*	*grandiclava*	(Y)		ET			PHL	3.0	Wichmann, 1912	Spessivftseff 1922, Balachowsky, 1949	Wood, 1982*; Kirkendall et al., 2015*	Synonym with *Pseudopolygraphus grandiclava* and *Pseudopolygraphus cembrae*
126	Scolytinae	Polygraphini	*Polygraphus*	*jezoensis*	(Y)	M	ET			PHL	2.8	Kerchev, 2015	Park, 2016	Kirkendall et al., 2015*;	
127	Scolytinae	Polygraphini	*Polygraphus*	*major*	(Y)	M	ET	M		PHL	3.2	Lyal and King, 1996	Khanday et al., 2020	Beaver and Browne, 1978; Kirkendall et al., 2015*	
128	Scolytinae	Polygraphini	*Polygraphus*	*nigrielytris*	Y	M	ET	M		PHL	2.7	Kerchev, 2020	Kerchev, 2020	Kirkendall et al., 2015*; Kerchev, 2020	
129	Scolytinae	Polygraphini	*Polygraphus*	*poligraphus*	(Y)		ET	HP	M	PHL	2.5	Wichmann, 1912	Lekander, 1959	Balachowsky, 1949; Kirkendall et al., 2015*; Belhabib et al, 2009; Rahmani et al., 2015	
130	Scolytinae	Polygraphini	*Polygraphus*	*proximus*	(Y)	M	ET	M	M	PHL	2.5	Kerchev, 2015; Kerchev, 2019	Kerchev, 2020; EFSA et al., 2020	Kerchev, 2014; Köbayashi and	

														Takagi, 2020; Takei et al., 2021	
131	Scolytinae	Polygraphini	*Polygraphus*	*rufipennis*	Y	M	ET	HP	B	PHL	2.5	Rudinsky et al., 1978a	Wood, 1982	Wood, 1982; Rudinsky et al., 1978a	
132	Scolytinae	Polygraphini	*Polygraphus*	*subopacus*	Y	M	ET	HP		PHL	1.9	Kerchev, 2020	Kerchev, 2020; Park, 2016	Kirkendall et al., 2015*; Kerchev, 2020	
133	Scolytinae	Scolytini	*Scolytus*	*abietis*	(Y)	B	GP	M	F	PHL	2.4	Equihua-Martinez and Furniss, 2009	Equihua-Martinez and Furniss, 2009	Wood, 1982	Synonym with *Scolytus opacus*
134	Scolytinae	Scolytini	*Scolytus*	*carpini*	(Y)		GP	M		PHL	2.3	Scholz, 1905 (in Barr, 1969)	Balachowsky, 1949	Balachowsky, 1949; Wood, 1982*; Kirkendall et al., 2015*; Petrov et al., 2019	
135	Scolytinae	Scolytini	*Scolytus*	*claviger*	(Y)			M		PHL	3.7	Wichmann, 1915	Park, 2016	Wood, 1982*; Kirkendall et al., 2015*; Petrov et al., 2019*	Synonym with *Eccoptogaster platystylus*
136	Scolytinae	Scolytini	*Scolytus*	*intricatus*	(Y)		GP	M		PHL	3.0	Gahan, 1900	Balachowsky, 1949	Balachowsky, 1949; Wood, 1982*; Kirkendall et al., 2015*; Petrov et al., 2019*	
137	Scolytinae	Scolytini	*Scolytus*	*laevis*	(Y)		GP	M		PHL	3.9	Scholz, 1905 (in Barr, 1969)	Petrov et al., 2019	Balachowsky, 1949; Wood, 1982*; Kirkendall et al., 2015*; Petrov et al., 2019*	
138	Scolytinae	Scolytini	*Scolytus*	*mali*	Y	B	GP	M	F	PHL	3.5	Rudinsky et al., 1978b	Grüne, 1979; Wood, 1982	Balachowsky, 1949; Wood, 1982	
139	Scolytinae	Scolytini	*Scolytus*	*multistriatus*	(Y)	B	GP	M	F	PHL	2.5	Jefferies and Fairhust, 1982	Wood, 1982	Wood, 1982	Bedoya et al., 2021 says that there is no sound production
140	Scolytinae	Scolytini	*Scolytus*	*pygmaeus*	(Y)		GP	M		PHL	2.0	Scholz, 1905 (in Barr, 1969)	Petrov et al., 2019	Balachowsky, 1949; Wood, 1982*; Kirkendall et al., 2015*; Petrov et al., 2019*	
141	Scolytinae	Scolytini	*Scolytus*	*ratzeburgii*	(Y)		GP	M		PHL	5.3	Gahan, 1900	Park, 2016	Balachowsky, 1949; Wood, 1982*; Kirkendall et al., 2015*; Petrov et al., 2019*	
142	Scolytinae	Scolytini	*Scolytus*	*rugulosus*	N			M	F	PHL	2.1	Bedoya et al., 2021; Gahan, 1900	Wood, 1982	Wood, 1982	
143	Scolytinae	Scolytini	*Scolytus*	*scolytus*	(Y)	B	GP	M	F	PHL	4.5	Jefferies and Fairhust, 1982	Balachowsky, 1949	Balachowsky, 1949	
144	Scolytinae	Scolytini	*Scolytus*	*ventralis*	Y	B	GP	M	F	PHL	3.2	Bedoya et al., 2021	Wood, 1982	Wood, 1982	
145	Scolytinae	Xyleborini	*Ambrosiodmus*	*obliquus*	N			IP	F	XYL	2.3	Bedoya et al., 2021	Wood, 1982	Wood, 1982*	Size taken from *Xyleborus obliquus* (Synonym)
146	Scolytinae	Xyleborini	*Cnestus*	*mutilatus*	N			IP	F	XYL	3.7	Bedoya et al., 2021	Schiefer and Bright, 2004	Osborn et al., 2023*	
147	Scolytinae	Xyleborini	*Dryoxylon*	*onoharaense*	N			IP	F	XYL	2.1	Bedoya et al., 2021	Bright and Rabaglia, 1999	Osborn et al., 2023*	
148	Scolytinae	Xyleborini	*Xyleborus*	*affinis*	N			IP	F	XYL	2.4	Bedoya et al., 2021	Wood, 1982	Wood, 1982*	
149	Scolytinae	Xyleborini	*Xyleborus*	*glabratus*	N			IP	F	XYL	2.0	Bedoya et al., 2021	Gomez et al., 2018	Brar et al., 2013	

150	Scolytinae	Xyleborini	*Xyleborinus*	*gracilis*	N			IP	F	XYL	1.8	Bedoya et al., 2021	Wood, 1982	Wood, 1982*	Synonym with *Xyleborus gracilis* and *Xyleborinus aspericauda*. Size taken from *Xyleborinus aspericauda* (Synonym)
151	Scolytinae	Xyleborini	*Xyleborinus*	*saxesenii*	N			IP	F	XYL	2.2	Bedoya et al., 2021	Wood, 1982	Wood, 1982	
152	Scolytinae	Xyleborini	*Xylosandrus*	*crassiusculus*	N			IP	F	XYL	2.5	Bedoya et al., 2021	Wood, 1982	Wood, 1982	
153	Scolytinae	Xyleborini	*Xylosandrus*	*germanus*	N			IP	F	XYL	2.2	Bedoya et al., 2021	Wood, 1982	Wood, 1982	
154	Scolytinae	Xyloterini	*Trypodendron*	*domesticum*	(Y)	M	VP	M	F	XYL	3.5	Klimetzek et al., 1981	Velez-Gavilan, 2022	Kirkendall et al., 2015*; Velez-Gavilan, 2022	
155	Scolytinae	Xyloterini	*Trypodendron*	*lineatum*	(Y)	M	VP	M	F	XYL	3.1	Klimetzek et al., 1981	Park, 2016	Kirkendall et al., 2015*; Kühnholz et al, 2021	
156	Scolytinae	Xyloterini	*Trypodendron*	*signatum*	(Y)	M	VP	M	F	XYL	3.2	Klimetzek et al., 1981	Makarov, 2005	Kirkendall et al., 2015*; Kühnholz, 2004	

S1 Table References

[1] Atkinson, T. H. *Doliopygus tenuis* (male) syntype *Crossotarsus tenuis* Strohmeyer, 1912 - USNM 53812, USNMENT 01547235. *Biodiversity Center, University of Texas at Austin* https://www.barkbeetles.info/amer_pix_target_species.php?lookUp=97 (2021).

[2] Balachowsky, A. S. Coléoptères Scolytides. *Faune de France* **50**, 1–320 (1949).

[3] Bain, J. *Pachycotes peregrinus* (Chapuis) (Coleoptera: Scolytidae). New Zealand Forest Service, Forest and Timber Insects in New Zealand **19** (1977).

[4] Barr, B. A. Sound production in Scolytidae (Coleoptera) with emphasis on the genus *Ips*. *Can. Entomol.* **101**(6), 636–672 (1969).

[5] Batra, L. R. Ecology of ambrosia fungi and their dissemination by beetles. *Trans. Kans. Acad. Sci.* (1903-) **66**(2), 213–236 (1963).

[6] Beaver, R.A. & Browne, F. G. The Scolytidae and Platypodidae (Coleoptera) of Penang, Malaysia. *Orient. Insects* **12**(4), 575–624 (1978).

[7] Beaver, R. A. & Liu, L. Y. A review of the genus *Genyocerus* Motschulsky (Coleoptera: Curculionidae: Platypodinae), with new synonyms and keys to species. *Zootaxa* **1576**, 25–56 (2007).

[8] Bedoya, C. L. Acoustic communication of bark and ambrosia beetles. (University of Canterbury, 2020).

[9] Bedoya, C. L., Brockerhoff, E. G., Hayes, M., Pawson, S. M., Najar-Rodriguez, A. & Nelson, X. J. Acoustic communication of the red-haired bark beetle (*Hylurgus ligniperda*). *Physiol. Entomol.* **44**(3-4), 252–265 (2019a).

[10] Bedoya, C. L., Nelson, X. J., Hayes, M., Hofstetter, R. W., Atkinson, T. H. & Brockerhoff, E. G. First report of luminous stimuli eliciting sound production in weevils. *Sci. Nat.* **106**(17), 1–4 (2019b).

[11] Bedoya, C. L., Hofstetter, R. W., Nelson, X. J., Hayes, M., Miller, D. R. & Brockerhoff, E. G. Sound production in bark and ambrosia beetles. *Bioacoustics* **30**(1), 58-73 (2021)

[12] Belhabib, R., Lieutier, F., Jamaa, M. L. B. & Nouira, S. Host selection and reproductive performance of *Phloeosinus bicolor* (Coleoptera: Curculionidae: Scolytinae) in indigenous and exotic Cupressus in Tunisia. *Can. Entomol.* **141**(6), 595–603 (2009).

[13] Brar, G. S., Capinera, J. L., Kendra, P. E., McLean, S. & Peña, J. E. Life cycle, development, and culture of *Xyleborus glabratus* (Coleoptera: Curculionidae: Scolytinae). *Fla. Entomol.* **96**(3), 1158–1167 (2013).

[14] Bright, D. Taxonomic monograph of the genus *Pityophthorus* Eichhoff in north and central America (Coleoptera: Scolytidae). *Mem. Ent. Soc. Can.* **113**(S118), 1–378 (1981).

[15] Bright, D. E. & Rabaglia, R. J. *Dryoxylon*, a new genus for *Xyleborus onoharaensis* Murayama, recently established in the southeastern United States (Coleoptera: Scolytidae). *Coleopt. Bull.* **53**, 333–337 (1999).

[16] Camacho-Pantoja, A. El género *Ips* (Coleóptera: Curculionidae: Scolytinae) en México. (Colegio de Postgraduados, 2012).

[17] Ceriani-Nakamurakare, E., Robles, C., González-Audino, P., Dolinko, A., Mc Cargo, P., Corley, J., Allison, J. & Carmarán, C. The Ambrosia Beetle *Megaplatypus mutatus*: A Threat to Global Broad-Leaved Forest Resources. *J. Integr. Pest. Manag.* **13**(1), 21 (2022).

[18] Chapuis, F. *Monographie des Platypides*. (Dessain, 1865).

[19] Deyrup, M. & Atkinson, T. H. Comparative biology of temperate and subtropical bark and ambrosia beetles (Coleoptera: Scolytidae, Platypodidae) in Indiana and Florida. *Gt. Lakes Entomol.* **20**(2), 59–66 (1987).

[20] Duvivier, A. 1891. Diagnoses de Coleoptères du Congo. *Annls Soc. Ent. Belg.* **35**, 376–377 (1891).

[21] European Food Safety Authority (EFSA), de la Peña, E., Kinkar, M. & Vos, S. Pest survey card on *Polygraphus proximus*. *EFSA Supporting Publications* **17**(1), 1780E (2020).

[22] Equihua-Martinez, A. & Furniss, M. M. Taxonomic status of *Scolytus opacus* and *Scolytus abietis* (Coleoptera: Curculionidae: Scolytinae): A comparative study. *Ann. Entomol. Soc. Am.* **102**, 597–602 (2009).

[23] Fabre, J. P. & Carle, P. Contribution à l'étude biologique d'*Hylurgus ligniperda* F.(Coleoptera Scolytidae) dans le Sud-est de la France. *Ann. Sci. For.* (*EDP Sciences)* **32**(1), 55–71 (1975).

[24] Fiala, T. & Holuša, J. The Bark Beetle *Phloeotribus rhododactylus* (Coleoptera: Curculionidae) Has a Stable Range in Europe. *Insects* **11**(12), 856(2020).

[25] Fleming, A. J., Lindeman, A. A., Carroll, A. L. & Yack, J. E. Acoustics of the mountain pine beetle (*Dendroctonus ponderosae*) (Curculionidae, Scolytinae): Sonic, ultrasonic, and vibration characteristics. *Can. J. Zool.* **91**, 235–244 (2013).

[26] Gahan, C. J. X. Stridulating Organs in Coleoptera. *Trans. R. Entomol. Soc. Lond.* **48**(3), 433–452 (1900).

[27] Gninenko, Y. I. *Xylechinus pilosus* Ratzeburg 1837 (Coleoptera, Curculionidae) – an important xylophage of Siberian fir. *Euroasian Entomol. J.* **19**(3), 131–133 (2020).

[28] Gomez, D. F., Rabaglia, R. J., Fairbanks, K. & Hulcr, J. North American Xyleborini north of Mexico: a review and key to genera and species (Coleoptera, Curculionidae, Scolytinae). *ZooKeys* **768**, 19–68 (2018).

[29] Grüne, S. Brief illustrated key to European bark beetles. (Verlag & Schaper, 1979)

[30] Hopkins, A. D. Contributions towards a monograph of the Scolytid beetles Part 1. The genus *Dendroctonus*. *Technical series of the U.S. Department of Agriculture - Bureau of Entomology* **17**, 35–51 (1909).

[31] Jefferies, D. & Fairhurst, C. P. Stridulatory organs of the elm bark beetles *Scolytus multistriatus* Marsham and *Scolytus scolytus* Fabricius. *J. Nat. Hist.* **16**(5), 759–762 (1982).

[32] Keeling, C. I., Bleiker, K. P. & Brooks, J. E. *Spruce Beetle: A Synthesis of Biology, Ecology, and Management in Canada* (eds K. P. Bleiker & J. E. Brooks) 53–66 (Natural Resources Canada, 2021).

[33] Kerchev, I. A. On monogyny of the four-eyed fir bark beetle *Polygraphus proximus* Blandf. (Coleoptera, Curculionidae: Scolytinae) and its reproductive behavior. *Entomol. Rev.* **94**, 1059–1066 (2014).

[34] Kerchev, I. A. Description of the stridulatory apparatus of the Far Eastern bark beetles *Polygraphus proximus* Blandford, 1894 and *P. jezoensis* Niisima, 1909 (Coleoptera, Curculionidae: Scolytinae). *Entmol. Rev.* **95**, 1191–1196 (2015).

[35] Kerchev, I. A. Context-Dependent Acoustic Signals in the Four-Eyed Fir Bark Beetle, *Polygraphus proximus* (Coleoptera: Curculionidae: Scolytinae). *Environ. Entomol.* **48**(1), 181–188 (2019).

[36] Kerchev, I. A. Interspecific differences of stridulatory signals in three species of bark beetles from the genus *Polygraphus* Er.(Coleoptera: Curculionidae, Scolytinae) inhabiting the island of Sakhalin. *PeerJ* **8**, e8281 (2020).

[37] Khanday, L., Buhroo, A., Kerchev, I., Singh, S. & Zubair, R M. A review of the Indian species of genus *Polygraphus* erichson, 1836 (Coleoptera: Curculionidae: Scolytinae) with bio-ecological notes on *P. major*, a pest of *Pinus wallichiana* A. B. Jacks (Pinaceae) in Kashmir, India. *Folia For. Pol. A* **62**, 171–183 (2020).

[38] Kirkendall, L. R. The evolution of mating systems in bark and ambrosia beetles (Coleoptera: Scolytidae and Platypodidae). *Zool. J. Linn. Soc.* **77**(4), 293–352 (1983).

[39] Kirkendall, L. R., Faccoli, M. & Ye, H. Description of the Yunnan shoot borer, *Tomicus yunnanensis* Kirkendall and Faccoli sp n. (Curculionidae, Scolytinae), an unusually aggressive

pine shoot beetle from southern China, with a key to the species of *Tomicus*. *Zootaxa* **1819**(1), 25–39 (2008).

[40] Kirkendall, L. R., Biedermann, H. & Jordal, B. H. Chapter 3 - Evolution and diversity of bark and ambrosia beetles. in *Bark beetles: biology and ecology of native and invasive species* (eds, Vega, F. & Hofstetter, R. W) 85–156 (Academic Press, 2015).

[41] Kleine, R. Der Stridulationsapparat der Ipidae 11. *Entomol. Bl. Biol. Syst. Käfer* **17**, 22–26 (1921).

[42] Klimetzek, D., Kiesel, K., Möhring, C. & Bakke, A. *Trypodendron lineatum*: reduction of pheromone response by male beetles. *Sci. Nat.* **68**(3), 149–151 (1981).

[43] Kobayashi, M. & Ueda, A. Preliminary study of mate choice in *Platypus quercivorus* (Murayama) (Coleoptera: Platypodidae). *Appl. Entomol. Zool.* **37**, 451–457 (2002).

[44] Köbayashi, K. & Takagi, E. Mating systems of the tree-killing bark beetle *Polygraphus proximus* (Coleoptera: Curculionidae: Scolytinae). *J. Insect Sci.* **20**(6), 38 (2020).

[45] Kühnholz, S. Chemical ecology and mechanisms of reproductive isolation in ambrosia beetles. (Simon Fraser University, 2004).

[46] Kühnholz, S., Gries, R. & Borden, J. H. Semiochemical-based Reproductive Isolation Among Sympatric Species of *Trypodendron* (Coleoptera: Curculionidae: Scolytinae). *Environ. Entomol.* **50**(1), 76–85 (2021).

[47] Labonte, J. & Takahashi, C. *Dactylotrypes longicollis* (Wollaston) (Coleoptera: Curculionidae: Scolytinae): An exotic bark beetle new to California and North America. *Pan-Pac. Entomol.* **88**, 222–230 (2012).

[48] Langor, D. W. & Hergert, C. R. Life history, behaviour, and mortality of the western ash bark beetle, *Hylesinus californicus* (Swaine) (Coleoptera: Scolytidae), in southern Alberta. *Can. Entomol.* **125**(5), 801–814 (1993).

[49] Lekander, B. Der doppeläugige fichtenbastkäfer *polygraphus poligraphus* L. *Meddelanden från Statens skogsforskningsinstitut* **48**(9), 1–127 (1959).

[50] Lewis, E. E., & Cane, J. H. Inefficacy of courtship stridulation as a premating ethological barrier for *Ips* bark beetles (Coleoptera: Scolytidae). *Ann. Entomol. Soc. Am.* **85**(4), 517–524 (1992).

[51] Lindeman, A. A. & Yack, J. E. What is the password? Female bark beetles (Scolytinae) grant males access to their galleries based on courtship song. *Behav. Process.* **11**, 123–131 (2015).

[52] Lindemann, K.E. Монографія короѣдовъ росски (Fam. Scolytidae lacord). *Извѣстія императорскаго общества любителей естествознанія, антропологій и этнографій.*

Monograph of the bark beetles of Russia (Fam. Scolytidae lacord). *Transactions of the imperial Society of Lovers of Natural History, Anthropology and Ethnography* **18** (1), 1–111. (1875).

[53] Liu, Y. B. & McLean, J. A. Observations on the biology of the ambrosia beetle *Gnathotrichus retusus* (LeConte)(Coleoptera: Scolytidae). *Can. Entomol.* **125**(1), 73–83 (1993).

[54] Loyining, M. K. & Kirkendall, L. R. Notes on mating system of *Hylesinus varius* (F.) (Col., Scolytidae), a putatively bigynous bark beetle. *J. Appl. Entomol.* **123**, 77–82 (1999).

[55] Lyal, C. H. C. & King, T. Elytro-tergal stridulation in weevils (Insecta: Coleoptera: Curculionoidea). *J. Nat. Hist.* **30**(5), 703–773 (1996).

[56] Lyon, R. L. A useful secondary sex character in *Dendroctonus* bark beetles. *Can. Entomol.* **90**, 582–584 (1958).

[56] Makarov, K. V. *Trypodendron signatum* F. (Scolytidae) - Moscow reg. Istrino distr. Pavlovskaya Sloboda 22.VI.2005. *Atlas of beetles of Russia* https://www.zin.ru/animalia/coleoptera/eng/trysigkm.htm (2005).

[57] Marcu, O. Beitrag zur Kenntnis der Stridulationsorgane bei Ipiden. *Zool. Anz.* **92**, 238–242 (1930d).

[58] Marcu, O. Ein neuer Beitrag zur Kenntnis der Stridulationsorgane bei Ipiden. *Zool. Anz.* **94**, 32–37 (1931).

[59] Maruthadurai, R., Desai, A. R. & Singh, N. P. First record of ambrosia beetle (*Euplatypus parallelus*) infestation on cashew from Goa, India. *Phytoparasitica* **42**, 57–59 (2014).

[60] Mercado-Vélez, J. E. & Negrón, J. F. Revision of the new world species of *Hylurgops* LeConte, 1876 with the description of a new genus in the Hylastini (Coleoptera: Scolytinae) and comments on some Palearctic species. *Zootaxa* **3785**(3), 301–342 (2014).

[61] McMullen, L., King, E. & Shenefelt, R. The oak bark beetle, *Pseudopityophthorus minutis*simus (Zimm.) (Coleoptera, Scolytidae) and its biology in Wisconsin. *Can. Entomol.* ***87***(11), 491–495 (1955).

[62] Menier, J. J. Existence d'appareils stridulatoires chez les Platypodidae (Coleoptera). *Ann. Soc. Entomol. Fr.* **12**, 347–353 (1976).

[63] Milligan, R. H. *Hylastes Ater* (Paykull),(Coleoptera: Scolytidae): Black Pine Bark Beetle. *Forest and Timber Insects in New Zealand* **29**, 1–8 (1978).

[64] Michalski, J. Stridulating organ as a secondary sexual character in *Ips sexdentatus* Boern (Coleoptera, Scolytidae). *Ann. Zool.* **20**, 27–33 (1925).

[65] Moucheron, B. Données complémentaires concernant le Scolyte *Xylechinus pilosus* (Ratzeburg, 1837) (Coleoptera, Curculionidae, Scolytinae). *Lambillionea* **116**(1), 74–76 (2016).

[66] Munro, J. W. The Genus *Hylastes*, Er., and its Importance in Forestry: a Study in Scolytid Structure and Biology. *Proc. R. Soc. Edinb.* **20**(3), 123–158 (1917).

[67] Nunberg, M. The stridulating organ of *Ips sexdentatus* Boern. (Col. Scolytidae). *Ann. Mus. Zool. Pol.* **14**, 135–140 (1950).

[68] Oester, P. T. & Rudinsky, J. A. Sound production in Scolytidae: stridulation by "silent" *Ips* bark beetles. *J. Appl. Entomol.* **79**, 421–427 (1975).

[69] Oester, P. T., Rudinsky, J. A., & Ryker, L. C. Olfactory and acoustic behavior of *Pseudohylesinus nebulosus* (Coleoptera, Scolytidae) on Douglas-fir bark. *Can. Entomol.* **113**, 645–650 (1981).

[70] Oester, P. T., Ryker, L. C. & Rudinsky, J. A. Complex male premating stridulation of the bark beetle *Hylurgops rugipennis* (Mann.). *Coleopt. Bull.* 93–98 (1978).

[71] Ohya, E. & Kinuura, H. Close range sound communications of the oak platypodid beetle *Platypus quercivorus* (Murayama) (Coleoptera: Platypodidae). *Appl. Entomol. Zool.* **36**(3), 317–321 (2001).

[72] Osborn, R. K., Castro, J., Duong ,T. A., Hulcr, J., Li, Y., Martínez, M. & Cognato, A. I. Symbiotic Fungi Associated With Xyleborine Ambrosia Beetles (Coleoptera: Curculionidae: Scolytinae) and the Imperative of Global Collaboration. *Ann. Entomol. Soc. Am.* **116**(1), 51–71 (2023).

[73] Park, S. W. Taxonomic review of Scolytinae and Platypodinae (Coeloptera: Curculionidae) in Korea. (Seoul National University, 2016).

[74] Petrov, A. V. A key to genera and species of the tribe Hylesinini Erichson, 1836 (Coleoptera: Curculionidae: Scolytinae) from Russia and adjacent countries. *Russ. Entomol. J.* **27**(2), 179–189 (2018)

[75] Petrov, A. V., Mandelshtam, M. Y., & Beaver, R. A. A key to species of the tribe Scolytini Latreille, 1804 (Coleoptera: Curculionidae: Scolytinae) from Russia and adjacent countries. *Russ. Entomol. J.* **28**(3), 286–302 (2019).

[76] Pfeffer, A. Fauna ČSR: Kůrovci – Scolytoidea. (in Czech) *Nakladatelství Československá Akademie věd* **324** (1955).

[77] Pfeffer, A. Taxonomischer Status von *Pityogenes bistridentatus* (Eichhoff) und die Schwarzkiefer (*Pinus nigra*) lebenden Borkenkäfer (Coleoptera, Scolytidae). *Acta Entomol. Bohemoslov.* **81**(4), 271–279 (1984).

[78] Phillips, T., Atkinson, T. & Foltz, J. Pheromone-based Aggregation in *Orthotomicus caelatus* (Eichhoff) (Coleoptera:Scolytidae). *Can. Entomol.* **121**(11), 933–940 (1989).

[79] Rahmani, R., Hedenström, E. & Schroeder, M. SPME collection and GC-MS analysis of volatiles emitted during the attack of male *Polygraphus poligraphus* (Coleoptera, Curcolionidae) on Norway spruce. *Z. Naturforsch. C* **70**(9-10), 265–273 (2015).

[80] Reay, S. D. Aspects of the ecology and behaviour of *Hylastes ater* (Paykull)(Coleoptera: Scolytidae) in second rotation *Pinus radiata* forests in the central North Island, New Zealand, and options for control. (University of Canterbury, 2000).

[81] Rudinsky, J. A. & Michael, R. R. Sound production in Scolytidae: Stridulation by female *Dendroctonus* beetles. *J. Insect Physiol.* **19**(3), 689–705 (1973).

[82] Rudinsky, J. A. & Vallo, V. The ash bark beetles *Leperisinus fraxini* and *Hylesinus oleiperda*: stridulatory organs, acoustic signals, and pheromone production. *J. Appl. Entomol.* **87**, 417–429 (1978).

[83] Rudinsky, J. A., Oester, P. T. & Ryker, L. C. Gallery Initiation and Male Stridulation of the Polygamous Spruce Bark Beetle *Polygraphus rufipennis*. *Ann. Entomol. Soc. Am.* **71** (3), 317–321 (1978a).

[84] Rudinsky, J. A., Vallo, V., & Ryker, L. C. Sound production in Scolytidae: Attraction and stridulation of *Scolytus mali* (Col., Scolytidae). *J. Appl. Entomol.* **86**, 381–391 (1978b).

[85] Rudinsky, J. A. Chemoacoustically induced behavior of *Ips typographus* (Col.: Scolytidae). *J. Appl. Entomol.* **88**, 537–54 (1979).

[86] Russo, G. V. Contributo alla conoscenza dei Coleotteri Scolitidi Fleotribo: *Phloeotribus scarabaeoides* (Bern.) Fauv. Parte prima. Morfologia, anatomia e istologia. *Bollettino del R. Laboratorio di Entomologia Agraria di Portici* **1**, 3–260 (1938).

[87] Ryker, L. C. Acoustic studies of *Dendroctonus* bark beetles. *Fla. Entomol.* **71**(4), 447–461 (1988).

[88] Schiefer, T. L. & Bright, D. E. *Xylosandrus mutilatus* (Blandford), an exotic ambrosia beetle (Coleoptera: Curculionidae: Scolytinae: Xyleborini) new to North America. *Coleopt. Bull.* **58**, 431–438 (2004).

[89] Scholz, R. Der Tonapparat von *Scolytus Ratzeburgi* Janson und die Entwicklung des Tonapparates bei einigen *Scolytus*-Arten. (Col.). *Insektenborse* **22**, 143–144 (1905).

[90] Schott, C. & Callot, H. J. Trois coléoptères scolytides nouveaux pour la faune de France observés en Alsace (*Xyleborus peregrinus* Eggers, *Lymantor aceris* Lindemann, *Phloeotribus caucasicus* Reitter (Col. Scolytidae). *Bulletin de la Société entomologique de Mulhouse JULSEP*, 67–70 (1994).

[91] Schwarz, E. A. A new scolytid beetle from tropical Florida. *Proceedings of the Entomological Society of Washington. Proc. Entomol. Soc. Wash.* **22**(8), 222–226 (1920).

[92] Sivalinghem, S. Acoustic communication in the pine engraver bark beetle, *Ips pini* (Coleoptera: Scolytinae). (Carleton University, 2011)

[93] Smith, G. D., Carroll, A. L. & Lindgren, B. S. Life history of a secondary bark beetle, *Pseudips mexicanus* (Coleoptera: Curculionidae: Scolytinae), in lodgepole pine in British Columbia. *Can. Entomol.* **141**(1), 56–69 (2009).

[94] Spessivtseff, P. Bestämningstabell över svenska barkborrar. *Meddelanden Från Statens Skogsförsöksansta*. A key to the sweedish bark beetles. *Communications From The State Forestry Research Institute* **19**(6), 454–492 (1922).

[95] Swedenborg, P., Jones, R., Ascerno, M. & Landwehr, V. *Hylurgopinus rufipes* (Eichhoff) (Coleoptera: Scolytidae): Attraction to broodwood, host colonization behavior, and seasonal activity in Central Minnesota. *Can. Entomol.* **120**(12), 1041–1050 (1988).

[96] Swedenborg, P., Jones, R. & Ryker, L. Stridulation and associated behavior of the native Elm bark beetle *Hylurgopinus rufipes* (Eichhoff) (Coleoptera: Scolytidae). *Can. Entomol.* **121**(3), 245–252 (1989).

[97] Takei S-y, Köbayashi K, Takagi E. Distribution pattern of entry holes of the tree-killing bark beetle Polygraphus *proximus*. *PLoS ONE* **16**(2), e0246812. (2021).

[98] Velez-Gavilan, J. *Trypodendron domesticum* (European hardwood ambrosia beetle). *CABI Compendium* **043**, 1–12 (2022).

[99] Vernoff, S. & Rudinsky, J. A. Sound production and pairing behavior of *Leperisinus californicus* Swaine and *L. oregonus* Blackman (Coleoptera: Scolytidae) attacking Oregon ash. *J. Appl. Entomol.* **90**, 58–74 (1980).

[100] Wichmann, H. Beitrag zur Kenntnis des Stridulationsapparates der Borkenkäfer. *Entomol. Bl. Biol. Syst. Käfer* **8**, 8–10 (1912).

[101] Wichmann, H. Zur Kenntnis der Ipiden. IV. *Entomol. Bl. Biol. Syst. Käfer* **11**, 213–217 (1915).

[102] Witkowski, R., Dyderski, M. K., Bełka, M. & Mazur, A. Potential European Geographical Distribution of *Gnathotrichus materiarius* (Fitch, 1858)(Coleoptera: Scolytinae) under Current and Future Climate Conditions. *Forests* **13**(7), 1097 (2022).

[103] Wood, S. L. A revision of the bark beetle genus *Dendroctonus* Erichson (Coleoptera: Scolytidae). *Great Basin Nat.* **23,** 1–117 (1963).

[104] Wood, S. L. The bark and ambrosia beetles of North and Central America (Coleoptera: Scolytidae), a taxonomic monograph. *Gt. Basin Nat. Mem.* **6**, 1–1359 (1982).

[105] Wood, S. L. New synonymy and new species of American bark beetles (Coleoptera: Scolytidae), Part XI. *Great Basin Nat.* **46**, 265–273 (1986).

[106] Yandell, K. L. Sound production of *Dendroctonus ponderosae* Hopkins (Coleoptera, Scolytidae): a comparison of populations from three host pines in Oregon. *J. Appl. Entomol.* **97**, 180–187 (1984).

[107] Ytsma, G. Stridulation in *Platypus apicalis*, *P. caviceps*, and *P. gracilis* (Col., Platypodidae). *J. Appl. Entomol.* **105**, 256–261 (1988).

[108] Yturralde, K. M. & Hofstetter, R. W. Characterization of stridulatory structures and sounds of the larger mexican pine beetle, *Dendroctonus approximatus* (Coleoptera: Curculionidae: Scolytinae). *Fla. Entomol.* **98**(2), 516–527 (2015).

[109] Žarković, I., Đuka, A., Franjević, M., Tomljanović, K., Papa, I., Krcivoj, T. & Hrašovec, B. Rare European beetle *Treptoplatypus oxyurus* (Coleoptera: Platypodidae) in managed uneven-aged forests of Croatia. *Forests* **13**(4), 580 (2022).

[110] Zervos, S. *Bispiculum inaequale* n. gen. & sp. (Nematoda: Tetradonematidae) from New Zealand wood-boring beetles (Curculionidae: Platypodinae), *N. Z. J. Zool.* **7**(2), 155–164 (1980).

[111] Zocchi, R. Insetti del Cipresso I. Il gen. *Phloeosinus* Chap. (Coleoptera Scolytidae) in Italia. *Redia* **41**, 129–225 (1956).
